# Supplementary material for: Early life adversity, contact with children’s social care services and educational outcomes at age 16 years: UK birth cohort study with linkage to national administrative records
Source: BMJ Open. 2019 Oct 7;9(10):e030213. doi: 10.1136/bmjopen-2019-030213 (PMC6797348; doi:10.1136/bmjopen-2019-030213)
Supplement: Supplementary data [file bmjopen-2019-030213supp001.pdf]

Early-life adversity, contact with children's social care services, and educational outcomes at age 16 years

Teyhan, Boyd, Wijedasa and Macleod

Supplementary Text - Additional details on some variables

- **Special Educational Needs (SEN)**

School Action (SA) - used when there is evidence that a child is not making progress at school and there is a need for action to be taken to meet learning difficulties. This can include involvement of extra teachers, use of different learning materials, special equipment or a different teaching strategy.

School Action Plus (SA+) - used where SA has not been able to help the child make adequate progress. The school has sought external services from the local education authority (LEA), the local health authority, or social services to help the child make adequate progress (e.g. speech and language therapist, educational psychologist etc.).

Statement of SEN - if the additional help provided by SA+ is not enough then the child's school or parents can apply to the LEA for a Statutory Assessment of the child's SEN in order to try and obtain a statement of SEN. The 'statement' is a document which sets out a child's SEN and any additional help that the child should receive. A Statement is normally made when all the educational provision required to meet a child's needs cannot reasonably be met by the resources within a child's school at SA+.

- **Early-life, maternal and family characteristics**

The following maternal, family and socioeconomic measures were reported by the mothers during their pregnancy with the study child via postal questionnaires. The categories for each variable are given in parenthesis following the variable name.

Highest educational qualification (university degree/A level; O level; vocational/none)

Early-life adversity, contact with children's social care services, and educational outcomes at age 16 years

Teyhan, Boyd, Wijedasa and Macleod

Financial Difficulties (quartiles of score with range 0–40, where 0 is no financial difficulties)

Housing tenure (owned/mortgaged; private rent; council rent; other)

Partner status (husband; live with partner; do not live with partner/no partner)

Smoking during pregnancy (no; yes)

Low social support - measured by response to 10 items with a low score defined as being in the bottom 10% (no; yes).

Maternal depression - measured by the Edinburgh Postnatal Depression Scale. Although this measure was originally designed for use with postnatal women, none of the 10 items is specific to this period and it has been validated for use at other times; it was chosen as it does not contain somatic items that could confound normal symptoms in pregnancy with depression (27). Quartiles were derived.

- **Proxy measures of socio-economic position when child aged 16**

The following measures were obtained from the National Pupil Database.

Child in receipt of free school meals (FSM) - eligibility is based on low parental income (28) (no; yes)

Child's residential neighbourhood deprivation - measured by the Income Deprivation Affecting Children Index (IDACI) (29), which gives the proportion of children (<0-15yrs) in a neighbourhood (lower super output area, average population 1500) who live in a low income family (<10%; 10 to <20%; 20 to <30%; 40%+).
