# Supplementary material for: Early life adversity, contact with children’s social care services and educational outcomes at age 16 years: UK birth cohort study with linkage to national administrative records
Source: BMJ Open. 2019 Oct 7;9(10):e030213. doi: 10.1136/bmjopen-2019-030213 (PMC6797348; doi:10.1136/bmjopen-2019-030213)
Supplement: Supplementary data [file bmjopen-2019-030213supp002.pdf]

Supplementary (online only) Tables

Table A: Summary of CLA and CIN data availability by year and period of birth

Table B: Summary of early-life (maternal and family) variables before and after multiple imputation

Table C: Summary of CIN data for ALSPAC participants linked to a post-2008 CLA record

Table D: Summary of care data for ALSPAC participants linked to a post-2005 CLA record

Table E: Comparison of care characteristics between ALSPAC CLA participants and CLA individuals in general population (in ALSPAC area and in England) of same age.

Table F: Educational attainment, absence, and special educational needs by care status – no SEN

Table G: Educational attainment, absence, and special educational needs by care status – no disability

Table A: Summary of CLA and CIN data availability by year and period of birth

	Expected school year in March of each year[1]					
Period of birth	2006[2]	2007[2]	2008[2]	2009[2,3]	2010[2,3]	2011[2,3]
April 1991 to August 1991	Year 10	**Year 11[4]**	Year 12	Year 13		
September 1991 to August 1992	Year 9	Year 10	**Year 11[4]**	Year 12	Year 13	
September 1992 to January 1993	Year 8	Year 9	Year 10	**Year 11[4]**	Year 12	Year 13

[1] The school year in England runs from September to July. In contrast, the CLA and CIN data collection year runs from April to March. For example, the 2006 CLA dataset would cover the period from April 2005 to March 2006; the oldest ALSPAC participants would have been in Year 10 in March 2006, and the youngest in Year 8.

[2] CLA data linked to NPD available

[3] CIN data linked to NPD available

[4] GCSE exams are taken in May/June of Year 11.

Determining who had CIN record during KS4:

For the CIN data, the youngest cohort participants were in Year 11 at the time of the earliest CIN data collection and they were coded as being ‘in need during KS4’ if they had a CIN record. However, the majority of the cohort were already in Year 12 or 13 at this time, and so we calculated the age they had been referred, and identified those who had been referred before they sat their KS4 exams (age on the 1st June of the year they were in Year 11 was used as a proxy for age that exams were taken).

Table B: Summary of early-life (maternal and family) variables before and after multiple imputation

Variables with missing data in study sample[1]	% missing	Categories[2]	Study sample [N=9545]	
			Before imputation [N specified for each variable individually]	Imputed [N=9545]
**Variables reported by mother during pregnancy**				
Relationship status	5.2		N=9048	
		Married (%)	75.7 (74.8-76.6)	74.7 (73.8-75.6)
		Resident partner (%)	16.1 (15.3-16.8)	16.5 (15.8-17.3)
		Non-resident/no partner (%)	8.2 (7.7-8.8)	8.7 (8.1-9.3)
Highest maternal education	9.7		N=8623	
		A Level or degree (%)	31.7 (30.8-32.7)	30.5 (29.5-31.4)
		O Level	36.8 (35.8-37.9)	36.5 (35.5-37.5)
		Vocational/none (%)	31.4 (30.4-32.4)	33.0 (32.0-34.0)
Financial difficulties	12.1		N=8387	
		Q1 (none) (%)	34.9 (33.9-35.9)	33.7 (32.7-34.7)
		Q4 (high) (%)	20.4 (19.5-21.3)	21.5 (20.6-22.4)
Housing tenure	5.7		N=9003	
		Owned/mortgaged (%)	74.6 (73.6-75.4)	73.4 (72.5-74.3)
Maternal smoking	5.0		N=9068	
		Yes (%)	26.2 (25.3-27.1)	26.9 (26.0-27.8)
Depression score	13.1		N=8294	
		Highest quartile (%)	22.7 (21.8-23.6)	23.5 (22.6-24.5)
Frequency of alcohol drinking in first trimester	6.0		N=8975	
		Never or <1 unit/week	84.8 (84.1-85.6)	84.8 (84.1-85.5)
		1-6 units or 7+ units/week	15.2 (14.4-15.9)	15.2 (14.5-15.9)
Any days drinking ≥4 units alcohol during second trimester	6.2		N=8950	
		Yes	16.8 (16.0-17.5)	16.9 (16.2-17.7)
Low social support	16.8		N=7942	
		Yes (%)	9.3 (8.7-10.0)	10.5 (9.8-11.2)
**Educational attainment from NPD**				
Capped percentage point score[3]	0.9	Mean	68.8 (68.4-69.2)	68.5 (68.1-68.9)

[1] The following variables had no missing data in study sample: child age, sex, maternal age at delivery, attainment of 5+ good GCSEs, persistent absence, SEN status, school attended.

[2] For brevity, not all categories are presented for each variable.

[3] The binary attainment variable (5+ good GCSEs) was complete for all those with a KS4 NPD record, but a small number (n=82) had missing capped point score data.

Table C: Summary of CIN data for ALSPAC participants linked to a post-2008 CIN record (but no CLA record)

Variable		CIN (no CLA) record N=209
Age (yrs) at referral date	Median (range)	16.7 (2.5-18.1)
Primary need status	Abuse or neglect (%, 95% CI)	22.0 (16.9-28.2)
	Child disability/illness (%, 95% CI)	23.4 (18.2-29.7)
	Parental illness/disability (%, 95% CI)	[n<5]
	Family in acute stress (%, 95% CI)	21.5 (16.4-27.7)
	Family dysfunction (%, 95% CI)	18.2 (13.5-24.1)
	Socially unacceptable behaviour (%, 95% CI)	[n<5]
	Low income (%, 95% CI)	[n<5]
	Absent parenting (%, 95% CI)	[n<5]
	Cases other than CIN (%, 95% CI)	[n<5]
	Not stated (%, 95% CI)	10.0 (6.6-15.0)
Child ever coded as having disability	Yes (%, 95% CI)	22.0 (16.9-28.2)

Table D: Summary of care data for ALSPAC participants linked to a post-2005 CLA record

		Linked to CLA record		
Variable		Overall<br><br>n=137	Eligible for one-third sample[1]<br>n=47	Ineligible for one-third sample[2]<br>n=90
Total number of periods of care[3]	Median, range	1 (1-13)	1 (1-8)	1 (1-13)
Total number of episodes of care[3]	Median, range	3 (1-28)	3 (1-24)	3 (1-28)
Age (yrs) at start of first period of care[3]	Median, range	13.2 (0-17.8)	11.5 (0-17.6)	13.7 (0.1-17.8)
Age (yrs) at end of last episode of care[3]	Median, range	17.7 (13.1-18.0)	18.0 (13.1-18.0)	17.1 (13.2-18.0)
		N=134	N=45	N=89
Total duration (days) in care [3,4]	Median, range	906 (1-5736)	1394 (2-5498)	604 (1-5736)
Primary need category at start of first period of care[3,5]	Abuse or neglect (%, 95% CI)	30.7 (23.4-39.0)	34.4 (21.6-49.1)	28.9 (20.3-39.3)
	Child disability (%, 95% CI)	16.1 (10.8-23.3)	19.1 (10.0-33.5)	14.4 (8.5-23.5)
	Parental illness/disability (%, 95% CI)[6]	4.4 (2.0-9.5)	/	/
	Family in acute stress (%, 95% CI)	18.2 (12.6-25.7)	17.0 (8.5-31.1)	18.9 (12.0-28.5)
	Family dysfunction (%, 95% CI)	17.5 (12.0-24.9)	25.5 (14.8-40.4)	13.3 (7.6-22.2)
	Socially unacceptable behaviour (%, 95% CI)[6]	8.8 (5.0-14.9)	/	/
	Absent parenting (%, 95% CI)[6]	4.4 (2.0-9.5)	/	/
Placement type for last episode of care	Foster care (%, 95% CI)	62.8 (54.3-70.5)	57.4 (42.6-71.1)	65.6 (55.0-74.8)
	Children's/residential/care home or school (%, 95% CI)	18.2 (12.6-25.7)	23.4 (13.2-38.1)	15.6 (9.3-24.8)
	Other (%, 95% CI)	19.0 (13.2-26.5)	19.1 (10.0-33.5)	18.9 (12.0-28.5)
Reason for last episode of care ending	Returned home to parents or relatives (%, 95% CI)	36.5 (28.8-45.0)	27.7 (16.5-42.6)	41.1 (31.3-51.7)
	Moved to independent living (%, 95% CI)	16.8 (11.4-24.1)	19.1 (10.0-33.4)	15.6 (9.3-24.8)
	Residential care funded by adult social services (%, 95% CI)	13.1 (8.3-20.0)	21.3 (11.6-35.8)	8.9 (4.4-17.0)
	Other (%, 95% CI)	32.8 (25.4-41.2)	31.9 (19.9-47.0)	34.4 (25.2-45.0)

[1]Individuals eligible for the one-third sample will have CLA records in the period 1998-2003 if they were looked-after during this time.
[2] No CLA data were collected from 2008-2003 for those ineligible for the one-third sample; therefore their looked-after status during this period is unknown.
[3] Measures relate to the data available from linkage only: these should be complete for those eligible for the one-third sample, but will not be for those in the ineligible sample who were looked-after during 1998-2003.
[4]The sum of the duration of all episodes of care, which may or may not have been consecutive.
[5]Refers to primary need at the start of the first period of care for which we have a record.
[6]Percentages not shown by one-third sample status for these need categories to prevent derivation of small cell counts (n<5).

Table E: Comparison of care characteristics between ALSPAC participants with CLA records and individuals with CLA records in general population of same age (in ALSPAC area and in England).

		Born 1991 or 1992 with CLA record in England[1]<br><br>n=43938	Born 1991 or 1992 with CLA record in ALSPAC area[1,2]<br><br>n=713	ALSPAC participants with CLA record<br><br>n=137
Total number of periods of care [3]	Median, range	1 (1-516)	1 (1-46)	1 (1-13)
Total number of episodes of care[3]	Median, range	2 (1-517)	2 (1-49)	3 (1-28)
Age (yrs) at start of first period of care[3]	Median, range	9.7 (0-18)	7.6 (0-18)	13.2 (0-17.8)
Age (yrs) at end of last episode of care[3]	Median, range	15.5 (0-19.9)	14.2 (0-18.2)	17.7 (13.1-18.0)
Last period of care ended <12yrs	Yes (%, 95% CI)	35.6 (35.2-36.1)	41.9 (38.4-45.6)	0
		n=43554	n=707	n=134
Total duration (days) in care [3,4]	Median, range	461 (1-6575)	427 (1-6069)	906 (1-5736)
		n=30250	n=453	n=137
Primary need category at start of first period of care[3,5]	Abuse or neglect (%, 95% CI)	35.3 (34.8-35.9)	26.3 (22.4-30.5)	30.7 (23.4-39.0)
	Child disability (%, 95% CI)	12.7 (12.4-13.1)	16.1 (13.0-19.8)	16.1 (10.8-23.3)
	Parental illness/disability (%, 95% CI)	3.9 (3.7-4.1)	5.1 (3.4-7.5)	4.4 (2.0-9.5)
	Family in acute stress (%, 95% CI)	10.9 (10.5-11.2)	18.5 (15.2-22.4)	18.2 (12.6-25.7)
	Family dysfunction (%, 95% CI)	12.4 (12.0-12.8)	20.5 (17.0-24.5)	17.5 (12.0-24.9)
	Socially unacceptable behaviour (%, 95% CI)	6.3 (6.1-6.6)	6.2 (4.3-8.8)	8.8 (5.0-14.9)
	Absent parenting (%, 95% CI)	18.1 (17.7-18.6)	7.3 (5.2-10.1)	4.4 (2.0-9.5)
		N=43602	N=712	N=137
Placement type for last episode of care	Foster care	32.8 (32.4-33.3)	42.0 (38.4-45.7)	62.8 (54.3-70.5)
	Children's/residential/care home/school	14.1 (13.7-14.4)	9.4 (7.5-11.8)	18.2 (12.6-25.7)
	Other	53.1 (52.6-53.6)	49.0 (44.9-52.3)	19.0 (13.2-26.5)
		N=39,647	N=644	N=137
Reason for last episode of care ending	Returned home to parents or relatives	21.9 (21.5-22.3)	24.8 (21.7-28.3)	36.5 (28.8-45.0)
	Moved to independent living	17.7 (17.4-18.1)	14.8 (12.2-17.7)	16.8 (11.4-24.1)
	Residential care funded by adult social services	4.8 (4.5-5.0)	7.6 (5.8-9.9)	13.1 (8.3-20.0)

[1]Excludes the 137 individuals identified as being in ALSPAC

[2]Includes only those in the care of one of the following local authorities: City of Bristol; Bath and North East Somerset; South Gloucestershire; North Somerset.

Table F: Educational attainment, absence, and special educational needs by care status excluding those with SEN (n=8145)

Outcome	Care status during KS4	Model 1[1] (Age and Sex)	Model 2[2] (KS4 variables)	Model 3[3] (Early-life variables)	Model 4[4] (Fully adjusted)
		OR (95% CI)	OR (95% CI)	OR (95% CI)	OR (95% CI)
5 A*-C GCSEs inc. Eng. & Maths	Not CIN or CLA	Ref	Ref	Ref	Ref
	CIN (not CLA)	0.04 (0.01-0.36)	0.08 (0.01-0.65)	0.08 (0.01-0.68)	0.11 (0.01-1.02)
	CLA	0.26 (0.09-0.77)	0.31 (0.10-0.98)	0.35 (0.11-1.10)	0.40 (0.12-1.31)
		Coeff (95% CI)	Coeff (95% CI)	Coeff (95% CI)	Coeff (95% CI)
Capped percentage score	Not CIN or CLA	Ref	Ref	Ref	Ref
	CIN (not CLA)	-24.1 (-31.8 to -16.5)	-10.6 (-17.6 to -3.6)	-18.0 (-25.0 to -10.9)	-8.6 (-15.2 to -2.0)
	CLA	-26.7 (-33.3 to -20.0)	-21.1 (-27.1 to -15.1)	-22.8 (-28.9 to -16.7)	-18.7 (-24.4 to -13.0)

[1]Adjusted for child age and sex

[2]Adjusted for child age and sex, plus KS4 time-point variables (persistent school absence, in receipt of free school meals, school mobility, IDACI of residential neighbourhood)

[3]Adjusted for child age and sex, plus early-life[maternal and SEP] variables (maternal age at delivery, education, partner status, housing tenure, financial difficulties, smoking, alcohol, depression, social support)

[4]Adjusted for child age and sex, plus KS4 and early life variables

Table G: Educational attainment, absence, and special educational needs by care status excluding those with a disability (n=9506)

Outcome	Care status during KS4	Model 1[1] (Age and Sex)	Model 2[2] (KS4 variables)	Model 3[3] (Early-life variables)	Model 4[4] (Fully adjusted)
		OR (95% CI)	OR (95% CI)	OR (95% CI)	OR (95% CI)
5 A*-C GCSEs inc. Eng. & Maths	Not CIN or CLA	Ref	Ref	Ref	Ref
	CIN (not CLA)	0.07 (0.02-0.25)	0.12 (0.03-0.43)	0.13 (0.04-0.45)	0.18 (0.05-0.65)
	CLA	0.17 (0.07-0.43)	0.19 (0.07-0.49)	0.28 (0.11-0.73)	0.29 (0.11-0.76)
		Coeff (95% CI)	Coeff (95% CI)	Coeff (95% CI)	Coeff (95% CI)
Capped percentage score	Not CIN or CLA	Ref	Ref	Ref	Ref
	CIN (not CLA)	-25.7 (-31.7 to -19.6)	-12.9 (-18.5 to -7.4)	-19.0 (-24.5 to -13.4)	-10.7 (-16.0 to -5.5)
	CLA	-28.6 (-34.2 to -23.0)	-24.6 (-29.7 to -19.5)	-22.8 (-28.0 to -17.6)	-20.8 (-25.7 to -16.0)

[1]Adjusted for child age and sex

[2]Adjusted for child age and sex, plus KS4 time-point variables (persistent school absence, in receipt of free school meals, school mobility, IDACI of residential neighbourhood)

[3]Adjusted for child age and sex, plus early-life[maternal and SEP] variables (maternal age at delivery, education, partner status, housing tenure, financial difficulties, smoking, alcohol, depression, social support)

[4]Adjusted for child age and sex, plus KS4 and early life variables
