# Supplementary material for: Early life adversity, contact with children’s social care services and educational outcomes at age 16 years: UK birth cohort study with linkage to national administrative records
Source: BMJ Open. 2019 Oct 7;9(10):e030213. doi: 10.1136/bmjopen-2019-030213 (PMC6797348; doi:10.1136/bmjopen-2019-030213)

Supplementary Figure 1: Partner, child and teacher questionnaire response rates by child social care status

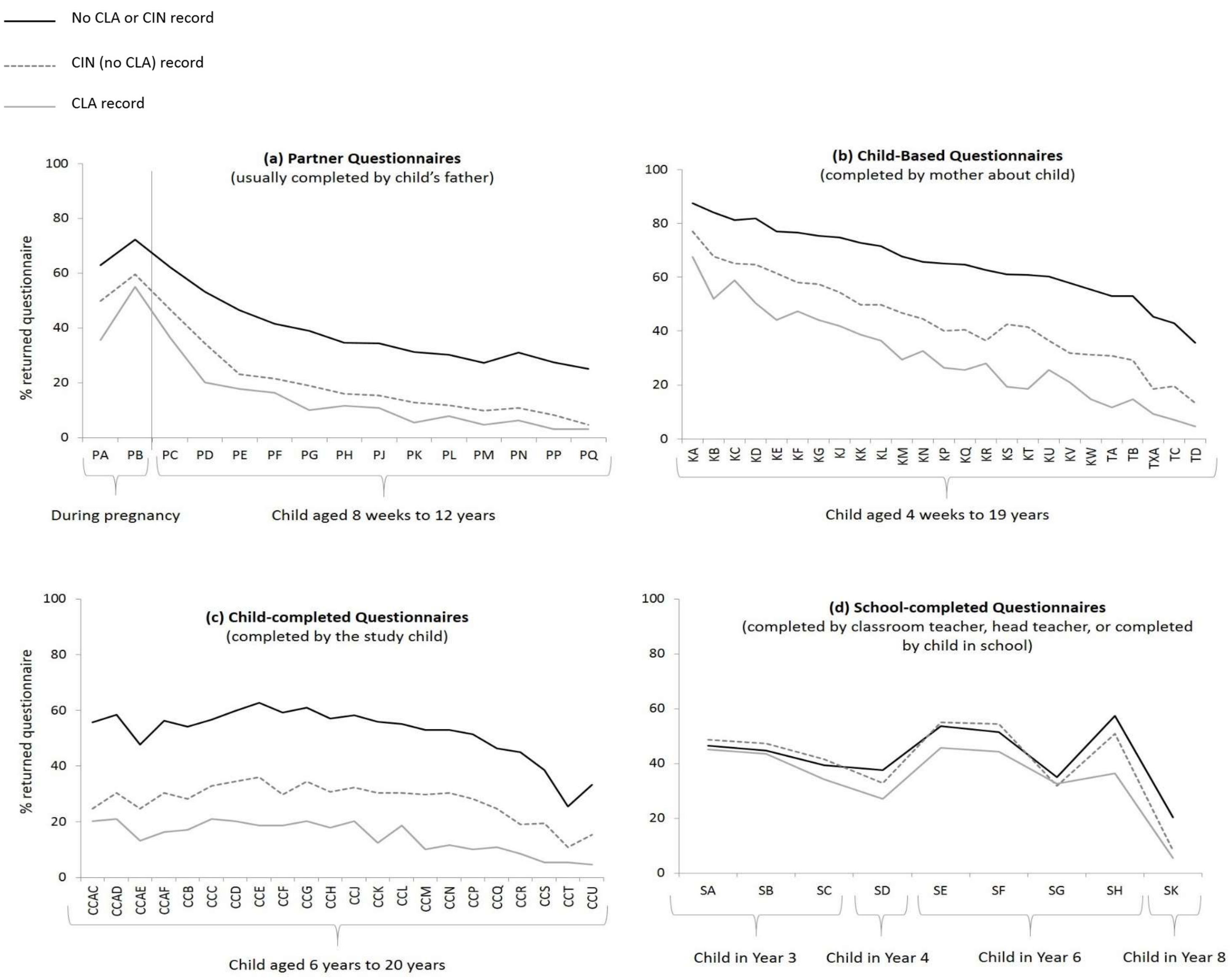

Supplement: Supplementary data [file bmjopen-2019-030213supp003.pdf]
