# Supplementary material for: Early life adversity, contact with children’s social care services and educational outcomes at age 16 years: UK birth cohort study with linkage to national administrative records
Source: BMJ Open. 2019 Oct 7;9(10):e030213. doi: 10.1136/bmjopen-2019-030213 (PMC6797348; doi:10.1136/bmjopen-2019-030213)
Supplement: Author's manuscript [file bmjopen-2019-030213.draft_revisions.pdf]

BMJ Open is committed to open peer review. As part of this commitment we make the peer review history of every article we publish publicly available.

When an article is published we post the peer reviewers' comments and the authors' responses online. We also post the versions of the paper that were used during peer review. These are the versions that the peer review comments apply to.

The versions of the paper that follow are the versions that were submitted during the peer review process. They are not the versions of record or the final published versions. They should not be cited or distributed as the published version of this manuscript.

BMJ Open is an open access journal and the full, final, typeset and author-corrected version of record of the manuscript is available on our site with no access controls, subscription charges or pay-per-view fees (http://bmjopen.bmj.com).

If you have any questions on BMJ Open's open peer review process please email
info.bmjopen@bmj.com

Early-life adversity, contact with children's social care services, and educational outcomes at age 16 years: UK birth cohort study with linkage to national administrative records

Journal:	*BMJ Open*
Manuscript ID	bmjopen-2019-030213
Article Type:	Research
Date Submitted by the Author:	05-Mar-2019
Complete List of Authors:	Teyhan, Alison; University of Bristol, Bristol Medical School, Population Health Sciences<br>Boyd, Andy; University of Bristol, Bristol Medical School, Population Health Sciences<br>Wijedasa, Dinithi; University of Bristol, Hadley Centre for Adoption and Foster Care Studies, School for Policy Studies<br>Macleod, John; University of Bristol, Bristol Medical School, Population Health Sciences
Keywords:	ALSPAC, record linkage, education, social care, looked-after, adolescence

SCHOLARONE™
Manuscripts

Early-life adversity, contact with children's social care services, and educational outcomes at age 16 years: UK birth cohort study with linkage to national administrative records

Alison Teyhan*[a], Andy Boyd[a], Dinithi Wijedasa[b], John Macleod[a]

*Corresponding author

Email: alison.teyhan@bristol.ac.uk

Phone: +44 (0)117 331 0058

[a] Population Health Sciences, Bristol Medical School, University of Bristol, Oakfield House, Oakfield Grove, Bristol, BS8 2BN, UK

[b] Hadley Centre for Adoption and Foster Care Studies, School for Policy Studies, University of Bristol, 8 Priory Road, Bristol, BS8 1TZ, UK

Word count: 3037

For peer review only

ABSTRACT (248 words)

Objectives: To use record linkage of birth cohort and administrative data to study educational outcomes of children who are looked-after (in public care) and in need (social services involvement), and examine the role of early life factors.

Setting, Design: Prospective observational study of children from the Avon Longitudinal Study of Parents and Children (ALSPAC), which recruited pregnant women in and around Bristol, UK in the early 1990s. ALSPAC was linked to the annual Children Looked-After (CLA) Data Return and Children In Need (CIN) Census. Educational outcomes at 16 years were obtained through linkage to the National Pupil Database. These included passing 5+ good GCSEs (grades A*-C, including English and Maths). Covariates included early-life adversity and social position.

Participants: 9545 children from ALSPAC who had complete education and covariate data were included in the main educational outcomes analyses.

Results: Overall, of the 12,868 ALSPAC participants linked to NPD data, 137 had a CLA record and a further 209 a CIN record during adolescence. These children were more disadvantaged than their peers and had little active study participation beyond infancy. In the main educational outcomes analyses, achievement of 5+ good GCSEs was low in the CLA (OR 0.14, 95% CI 0.05-0.35) and CIN (0.11, 0.05-0.27) groups relative to their peers. Measured early-life factors explained little of this difference.

Conclusions: Data linkage enabled the study of educational outcomes in children with social services contact. These children had substantially worse educational outcomes relative to their peers, for reasons likely to be multifactorial.

Key Words: ALSPAC; record linkage; education; social care; looked-after; adolescence

For peer review only - http://bmjopen.bmj.com/site/about/guidelines.xhtml

Article Summary - Strengths and Limitations

- We link a population-based birth cohort study (ALSPAC) to social care and educational records, and demonstrate that record linkage offers a means to identify vulnerable children in a cohort and increase their inclusion in research.
- The children in ALSPAC who had been looked-after (in public care) were broadly representative in terms of their care characteristics of children nationally of the same age who had been looked-after.
- We were only able to identify children who had been in care or in need during adolescence.
- Cohort data availability for children with social care records in adolescence was low beyond infancy.

For peer review only

INTRODUCTION

Children with social services contact, including those in public care, are at higher risk of poor outcomes than their peers, including low educational attainment, substance abuse, and mental illness(1-10). The extent to which this reflects early-life adversity prior to contact with services as opposed to later influences is unclear. Outcomes mainly resulting from early adversity may be less amenable to change through social care interventions, requiring alternative prevention strategies. These children are challenging to study using traditional research methods. A recent Children's Commissioner for England report highlights that vulnerable children are 'absent or poorly measured in national studies'(11), and children's social care is a difficult area in which to conduct randomised controlled trials(12). Further, those who experience extreme adversity are likely under-represented in birth cohort studies due to low recruitment and high attrition, and identification of vulnerable children is challenging due to reliance on parental-report.

Children with social services contact in England do however have high levels of administrative data. The term 'in need' refers to children who have been referred to and assessed by social services and found to be 'unlikely to achieve or maintain a reasonable level of health or development, or whose health and development is likely to be significantly or further impaired, without the provision of services; or a child who is disabled'(13). Almost 390,000 children are currently classified as 'in need'(14). Some children in need may enter the public care system and become a 'looked-after' child. Presently over 72,000 children are looked-after(15), with the majority placed with foster carers(1).

While routine statistics using social care data can highlight poor outcomes, e.g. low average educational attainment, they lack information on early-life and family characteristics(1, 16, 17). These types of data are readily available in birth cohort studies. Linking cohort data to

For peer review only - http://bmjopen.bmj.com/site/about/guidelines.xhtml

social care records could therefore provide a means of identifying children in need and looked-after without reliance on parental-report. Further, using additional linked data to measure outcomes potentially enables the child's inclusion in analyses even if their family have stopped actively participating in the cohort study.

We use record linkage to a birth cohort to examine the effect of being in need or looked-after in adolescence on educational outcomes at age 16 years: the low attainment of many in need and looked-after children at this age is a concern as it can compound their disadvantaged childhoods to limit future education, employment, and general life chances(18).

METHODS

Data

Avon Longitudinal Study of Parents and Children (ALSPAC)

Pregnant women living in and around the city of Bristol, UK with expected date of delivery April 1991 to December 1992 were eligible to participate in ALSPAC. There were 14,541 pregnancies enrolled, resulting in 13,988 children alive at one year, including 13,972 singletons and twins. This 'core sample' was later bolstered by further eligible children: an additional 713 from age 7-18yrs, and to date 183 since age 18yrs. The mothers, their partners, and the study children are studied via questionnaires and clinic visits. Teachers also completed questionnaires on the children. Further details are provided in cohort profiles(19, 20) and searchable data-dictionary(21). For the main analyses on educational outcomes, the sample was restricted to: core, one child per family, with education data (n=9545, Figure 1).

Ethical approval for ALSPAC was obtained from the ALSPAC Ethics and Law Committee and Local Research Ethics Committees. When study children reached age 18, they were sent 'fair processing' materials which described ALSPAC's intended use of their health and

For peer review only - http://bmjopen.bmj.com/site/about/guidelines.xhtml

administrative records, and gave a clear means to object(22). Education data were not extracted for participants who objected, or who were not sent fair processing materials.

Linkage data

Data on children who are looked-after, or have been referred as a child in need, are collected annually via the Children Looked-After (CLA) Data Return(23) and the Children in Need (CIN) Census(24). The CIN Census covers all children referred to children's social services even if no further action is taken. The CLA Return and the CIN Census have been linked to the National Pupil Database (NPD), a repository of education data for schools in England(25), since their 2005/06 and 2008/09 data collections respectively. ALSPAC has an established link to the NPD, and thus to any post-2005 CLA or post-2008 CIN record for participants in the NPD. Earlier CLA records were also obtained for those with a post-2005 record. However, CLA data collection was only on a random one-third sample of looked-after children from 1998-2003, meaning no records exist for many looked-after children in this period(23). Insufficient identifiers exist within the CLA dataset to enable linkage of ALSPAC to pre-2005 CLA records for those without a post-2005 record.

We also obtained CLA records for all individuals in the CLA Return of a similar age (born January 2001-December 2002) to form two comparison groups: (1) ever looked-after in England (n=43,938); (2) ever looked-after in the four local authorities that approximate the ALSPAC recruitment area (Bristol City; South Gloucestershire; North Somerset; Bath and North East Somerset) (n=713).

Measures

Educational outcomes

Pupils in England study General Certificate of Education (GCSE) courses during Key Stage 4 (KS4) of their education (Years 10 and 11, aged 14-16yrs) and take GCSE exams at the end

For peer review only - http://bmjopen.bmj.com/site/about/guidelines.xhtml

of Year 11. The oldest ALSPAC children sat their GCSE exams in 2007, the youngest in 2009. Our main outcomes were two measures of attainment. First, a binary measure: achievement of 5+ good GCSEs (grades A*-C, including English and Maths). Second, a continuous measure: capped point score, expressed as a percentage of the maximum possible capped point score (based on the eight best grades obtained, with each grade assigned a numerical value)(26). Secondary educational outcomes included: persistent absence (≥10% of half days); special educational needs (SEN) status (see footnote of Table 2); and school mobility (whether child joined school during KS4).

Contact with children's social care services

Contact with children's social care services (referred to as 'social care status' hereafter) was summarised in two variables. The first specified whether a child had any post-2005 CLA record(s) or post-2008 CIN record(s) (i.e. was looked-after or referred to social care services at any time for which we have linked social care data). The second summarised social care status during KS4 only. This restriction was necessary for the educational outcomes analyses to ensure our exposure preceded our outcome, plus these are the only two school years with CLA data coverage for all children in our sample (Supplementary Table A). By definition children who are looked-after are also in need but we use 'in need' to refer to children with a CIN but not a CLA record. The reference group comprised children with a KS4 record in the NPD who had no linked social care record.

Variables related to being in care or in need were derived from the linked data as follows. CIN Census: category of need; age referred. CLA Return: category of need; age first period of care (POC) started (POC is a period of time when child is continuously looked-after by the local authority); number of POC and episodes of care (a POC is comprised of 'episodes', each representing a period of being looked-after under the same legal status and in the same

For peer review only - http://bmjopen.bmj.com/site/about/guidelines.xhtml

placement); placement type (foster; children's home/residential home/residential school; other [no further disaggregation possible due to small numbers]).

Covariates

These included child age and sex, plus measures related to family socio-economic position (SEP). Early-life exposures were reported by the mother during pregnancy: age at delivery; highest educational qualification; financial difficulties; housing tenure; partner status; smoking; social support; depressive symptoms(27). Later measures of SEP (during KS4) were obtained from the NPD: receipt of free school meals (FSM)(28); and child's residential neighbourhood deprivation measured by the Income Deprivation Affecting Children Index (IDACI)(29). More details in Supplementary Text.

Statistical analyses

Descriptive statistics were used to: summarise the social care data linked to ALSPAC children; compare the ALSPAC looked-after sample to the two non-ALSPAC looked-after comparison groups; compare child, maternal and SEP characteristics by social care status; describe questionnaire completion rates by social care status.

Associations between social care status and educational outcomes were examined using multilevel regression models (individual level 1, school level 2). Linear models were used for capped point score, logistic for attainment of 5+ good GCSEs. Models adjusted for age and sex (Model 1), then also for KS4 measures [FSM, neighbourhood deprivation, school mobility] (Model 2), or for early-life exposures (Model 3). We then adjusted for all KS4 and early-life variables (Model 4). Multiple imputation using chained equations was used to impute missing data (supplementary Table B) for the educational outcomes analyses sample (n=9545). 100 datasets were imputed.

For peer review only - http://bmjopen.bmj.com/site/about/guidelines.xhtml

In sensitivity analyses, models were restricted to children with no SEN (n=8145) or no disability (n=9506). Social care status at any time was also considered. Finally, we described associations between social care characteristics (e.g. placement type, reason for being in need) and capped point score in those with CIN or CLA records: to maximise sample size, we included all those who had these records at any time and who had capped point score data.

Patient and Public Involvement

Patients, the public, and study participants were not directly involved in this study. Some ALSPAC participants are members of a committee which meets bi-monthly to provide insights and advice on general ALSPAC study design, methodology and acceptability for participants.

RESULTS

Children in ALSPAC with social care records

Of those with a post-2008 CIN (but no CLA) record (n=209) the most common needs at referral were child disability, abuse or neglect, and family in acute stress. Of those with a post-2005 CLA record (n=137), the most common primary need was abuse or neglect (Supplementary Tables C and D). Median total time in care was 2.6 years. Foster care was the most common placement type.

Comparison to non-ALSPAC looked-after children

The ALSPAC children with CLA records were generally similar to those of children born at the same time who were ever in care in England (comparison group 1) or in the area in and around Bristol (comparison group 2) in terms of primary need (Supplementary Table E). Importantly, many of those who had ever had a CLA record in the two comparison groups

For peer review only - http://bmjopen.bmj.com/site/about/guidelines.xhtml

(36% of group 1; 42% of group 2) had left care before the age of 12 (the youngest age at which we were able to link CLA records to ALSPAC).

Availability of cohort data

Maternal questionnaire response rates were highest for participants with no social care record and lowest for those with a CLA record at all time points. Differences generally widened over time (Figure 2). Patterns were similar for partner and child, but not teacher, questionnaires (supplementary Figures 3a-d).

Educational outcomes at 16 years

Of the 9545 children in these analyses, 49 had CLA and 64 CIN (no CLA) records during KS4. These groups were more disadvantaged than their peers in early-life and during KS4 (Table 1). They were more likely to have joined their school recently.

Of those with CIN or CLA records, <15% passed 5+ good GCSEs compared to >50% of their peers. Mean percentage scores were also markedly lower (Table 2). They were more likely to have SEN and persistent absence rates were higher, particularly for the in need group. Adjustment for school absence, neighbourhood deprivation, and receipt of FSM attenuated associations slightly for the CIN group but had less of an impact for the CLA group (Table 3). Adjustment for early-life maternal and SEP factors had more of an attenuating effect for the CLA than the CIN group. Attainment differences between these groups and their peers remained in the fully adjusted model.

In sensitivity analyses, when social care records at any time were considered, patterns were similar for the CLA group (n=76), while the CIN group (n=148) tended to do better than when restricted to only those who were in need during KS4 (Table 2). When the sample excluded those with SEN or disability, results were similar to those of the main analyses (supplementary tables F and G).

For peer review only - http://bmjopen.bmj.com/site/about/guidelines.xhtml

Estimates of the relationship between social care characteristics and attainment were imprecise due to small numbers. Those in foster placements had higher capped percentage scores (mean 35.9, 95% CI 29.3 to 42.5, n=60) than those in children's/residential homes/residential schools (25.0, 12.3 to 37.8, n=12). With regards need status, for both the CLA and CIN groups, 'child disability' was associated with the lowest attainment, and 'parental illness/disability' the highest but confidence intervals were wide and overlapping.

For peer review only

For peer review only - http://bmjopen.bmj.com/site/about/guidelines.xhtml

Table 1: Summary of maternal, family and child characteristics, by social care status of child during Key Stage 4

		Child's social care status during KS4		
		No CLA/CIN record n=9432	CIN (no CLA) record n=64	CLA record n=49
**Maternal and family characteristics during pregnancy[1]**		% (95% CI)		
Maternal age (at delivery)	<=23 years	18.4 (17.7-19.2)	39.1 (27.7-51.8)	28.6 (17.4-43.2)
	>33 years	12.3 (11.6-12.9)	7.8 (3.2-17.8)	14.3 (6.8-27.6)
Relationship status	Married	75.0 (74.1-75.9)	53.7 (40.5-66.8)	49.8 (34.6-65.1)
	Resident partner	16.5 (15.7-17.3)	16.7 (6.5-26.9)	27.3 (13.3-41.3)
	Non-resident/no partner	8.5 (7.9-9.1)	29.6 (17.5-41.7)	22.9 (9.6-36.2)
Highest maternal education	A Level or degree	30.7 (29.7-31.7)	10.8 (2.5-19.1)	11.4 (1.1-21.8)
	O Level	36.5 (35.5-37.5)	40.2 (26.8-53.7)	26.1 (11.2-40.9)
	Vocational/none	32.8 (31.8-33.8)	48.9 (35.2-62.7)	62.5 (46.4-78.6)
Financial difficulties	Highest quartile	21.2 (20.3-22.1)	41.7 (27.8-55.7)	46.2 (29.3-63.2)
Housing tenure	Owned/mortgaged	73.7 (72.8-74.6)	54.1 (41.2-67.1)	33.2 (18.5-48.0)
Maternal smoking	Yes	26.6 (25.7-27.6)	41.6 (28.2-55.0)	59.8 (43.5-76.2)
Depression score	Highest quartile	23.3 (22.4-24.3)	29.5 (16.5-42.6)	48.4 (32.3-64.5)
Low social support	Yes	10.3 (9.6-11.0)	21.3 (9.0-33.6)	26.8 (11.2-42.4)
**Child, school and neighbourhood characteristics during KS4[1]**		% or mean (95% CI)		
Sex	Female	49.6 (49.4-51.4)	51.6 (39.1-63.8)	49.0 (36.8-65.1)
Age at start of Year 11	Mean (years)	15.5 (15.4-15.5)	15.5 (15.4-15.6)	15.5 (15.4-15.5)
In receipt of free school meals	Yes	6.1 (5.6-6.6)	26.6 (17.0-39.0)	10.2 (4.2-22.9)
Joined school during KS4	Yes	1.4 (1.2-1.6)	7.8 (3.2-17.8)	12.2 (5.4-25.3)
Neighbourhood deprivation (IDACI)	Low, <10%	43.9 (42.7-44.9)	20.3 (12.0-32.3)	28.6 (17.4-43.2)
	High, >=40%	10.1 (9.5-10.8)	25.0 (15.7-37.4)	20.4 (11.1-34.5)

[1] For brevity, not all categories are presented for each categorical variable.

For peer review only - http://bmjopen.bmj.com/site/about/guidelines.xhtml

Table 2: Educational attainment, persistent absence, and special educational needs by child social care status

	Social care status during KS4			Social care status any time		
	No CLA/CIN record n=9432	CIN (no CLA) record n=64	CLA record n=49	No CLA/CIN record n=9321	CIN (no CLA) record n=148	CLA record n=76
**Educational Attainment**	% or mean (95% CI)			% or mean (95% CI)		
5+ A*-C GCSEs including English & Maths	53.0 (52.0-54.0)	10.9 (5.2-21.6)	12.2 (5.4-25.3)	53.3 (52.3-54.4)	19.6 (13.9-26.9)	10.5 (5.3-19.9)
Capped percentage point score	68.9 (68.5-69.3)	37.4 (31.3-43.4)	34.9 (27.4-42.3)	69.1 (68.7-69.5)	47.7 (43.7-51.7)	33.9 (28.2-39.5)
**Special Educational Needs (SEN)**						
School action[1]	8.5 (8.0-9.1)	12.5 (6.3-23.4)	n<5	8.4 (7.9-9.0)	16.2 (11.1-23.1)	9.2 (4.4-18.4)
School action plus[2]	3.1 (2.8-3.5)	15.6 (8.5-27.0)	24.5 (14.2-38.9)	3.1 (2.7-3.4)	8.1 (4.6-13.8)	21.1 (13.2-31.9)
Statement of Special Educational Needs[3]	2.4 (2.1-2.8)	46.9 (34.8-59.4)	24.5 (14.2-38.9)	2.3 (19.8-2.6)	22.3 (16.2-29.8)	35.5 (25.4-47.1)
**Persistent absence**	6.8 (6.3-7.3)	32.8 (22.2-45.5)	18.4 (9.6-32.3)	6.7 (6.2-7.2)	19.6 (13.9-26.9)	21.1 (13.2-31.9)

[1] School Action (SA) is used when there is evidence that a child is not making progress at school and there is a need for action to be taken to meet learning difficulties. This can include involvement of extra teachers, use of different learning materials, special equipment or a different teaching strategy.

[2] School Action Plus (SA+) used where SA has not been able to help the child make adequate progress. The school has sought external services from the local education authority (LEA), the local health authority, or social services to help the child make adequate progress (e.g. speech and language therapist, educational psychologist etc.).

[3] If the additional help provided by SA+ is not enough then the child's school or parents can apply to the LEA for a Statutory Assessment of the child's SEN in order to try and obtain a Statement of SEN. The 'statement' is a document which sets out a child's SEN and any additional help that the child should receive. A Statement is normally made when all the educational provision required to meet a child's needs cannot reasonably be met by the resources within a child's school at SA+.

For peer review only

Table 3: Association between child social care status and educational outcomes in adjusted models

Attainment Outcome	Care status during KS4	Model 1[1] (Age and Sex)	Model 2[2] (KS4 variables)	Model 3[3] (Early-life variables)	Model 4[4] (Fully adjusted)
		OR (95% CI)	OR (95% CI)	OR (95% CI)	OR (95% CI)
5+ A*-C GCSEs including English & Maths	Not CIN or CLA	Ref	Ref	Ref	Ref
	CIN (not CLA)	0.11 (0.05-0.27)	0.17 (0.07-0.40)	0.15 (0.06 to 0.36)	0.19 (0.08-0.46)
	CLA	0.14 (0.05-0.35)	0.14 (0.06-0.36)	0.25 (0.10 to 0.63)	0.24 (0.09-0.63)
		Coeff (95% CI)	Coeff (95% CI)	Coeff (95% CI)	Coeff (95% CI)
Capped percentage point score	Not CIN or CLA	Ref	Ref	Ref	Ref
	CIN (not CLA)	-22.1 (-26.8 to -17.5)	-14.1 (-18.4 to -9.9)	-18.5 (-22.8 to -14.2)	-13.2 (-17.3 to -9.2)
	CLA	-28.4 (-33.5 to -23.2)	-25.0 (-29.7 to -20.3)	-21.8 (-26.5 to -17.1)	-20.4 (-24.9 to -16.0)

[1]Adjusted for child age and sex
[2]Adjusted for child age and sex, plus KS4 time-point variables (persistent school absence, in receipt of free school meals, school mobility, IDACI of residential neighbourhood)
[3]Adjusted for child age and sex, plus early-life [maternal and SEP] variables (maternal age at delivery, education, partner status, housing tenure, financial difficulties, smoking, depression, social support)
[4]Adjusted for child age and sex, plus KS4 and early life variables

For peer review only

DISCUSSION

Children who were looked-after or in need during KS4 had low attainment at age 16. Measured early-life exposures were not a major explanatory factor. We believe this is the first time linkage to the CLA Return and CIN Census has been used to identify birth cohort participants who were looked-after or in need during adolescence. As linkage data were also used for outcome measures, participants could be included even if their families no longer actively participated in the cohort study. Record linkage therefore allowed vulnerable children to not only be included in research but to be the focus of it. However, the identification and inclusion of in need and looked-after children in research using record linkage does have challenges.

For cohort studies in England with relevant permissions, linkage to the CLA Return and CIN Census via the NPD offers a convenient means of identifying participants who have been in need or looked-after. For cohorts younger than ALSPAC, this method would allow identification of social care records that cover most, if not all, of participants' childhoods. However, in ALSPAC we were only able to link to records covering a period during adolescence. Consequently, outcomes at younger ages cannot be examined by social care status in ALSPAC using this method. Of the looked-after children in England the same age as the ALSPAC participants, we found around 40% had left the care system by the age of 12. Consequently, our reference group likely includes children who were looked-after or in need at younger ages only.

Examination of questionnaire response rates showed the value of using linked outcome data to increase the inclusion of vulnerable children in research: there was little questionnaire data available beyond infancy for participants with social care records in adolescence. In this current study, we examined educational outcomes at age 16, obtained from the NPD. The

For peer review only - http://bmjopen.bmj.com/site/about/guidelines.xhtml

association between social care status and other later outcomes available from linked data could also be investigated using ALSPAC, such as mental illness or entry into higher education.

ALSPAC participants with CLA and CIN records in adolescence had lower educational attainment than their peers in the reference group. In the most recent national data available, attainment patterns by social care status broadly reflect these findings(1). We found persistent absence rates to be considerably lower for those looked-after than those in need during KS4. Similarly, in the national data (on pupils of all ages) 9% of looked-after children were persistent absentees and 28% of children in need(1). Therefore, although our participants were in KS4 around 10 years ago and the number with social care records small, the patterning of educational characteristics by care status is broadly similar to the present-day situation.

Using both ALSPAC questionnaire data and measures from the NPD, we found a persistence of disadvantage from early-life to adolescence for participants with CIN and CLA records. Social disadvantage is known to be strongly associated with poorer educational attainment(30, 31), and our SEP measures were strongly related to the educational outcomes. Adjustment for them attenuated associations slightly but the low attainment of the CLA and CIN groups remained. We are not considering the SEP measures as confounders but rather part of the complex causal pathway from early-life adversity through to poor educational attainment. Little of the poor educational outcomes in the looked-after and in need groups appeared to be explained by the early-life exposures we considered, suggesting there is scope for later experiences, including social care, to improve outcomes.

While aspects of care itself could be important contributors to educational outcomes, ascertaining direction of causality in the relationship between child behaviours, care

For peer review only - http://bmjopen.bmj.com/site/about/guidelines.xhtml

characteristics, and educational outcomes is difficult. As expected, we found children in foster care had higher attainment than those not in family-based care: the latter children are likely to be those whose foster placements have broken down, reflecting complex additional needs and challenging behaviours. Further, foster carers may have greater commitment and longer-term interest in the child than group care staff(32). We were unable to consider placement stability, which has previously been shown to be beneficial(33). However, in concordance with previous studies, school mobility was associated with lower attainment(34, 35) and children with CLA or CIN records were much more likely to have changed school during KS4 than their peers.

The relatively high proportion of looked-after and in need children with SEN or disability did not appear to explain the low average attainment of these groups. Similarly, in the national data looked-after children with no identified SEN made less educational progress than non-looked-after children(1). It is important to note that the attainment gap between looked-after and in need children and their peers is apparent from a young age, often before the child enters care(1, 33). Being looked-after may not be the principal cause of poor attainment, rather it is a marker of extreme childhood adversity, which is itself associated with poor outcomes. Being in care is often beneficial for a child's education(17, 18, 33).

Strengths of this study include the use of a novel method to identify vulnerable adolescents in a population-based cohort, and objective outcome measures. Limitations include incomplete ascertainment of social care record status, little cohort data beyond early childhood for those with social care records, and small numbers. Children who experience the most disadvantaged starts in life are likely under-represented in ALSPAC as their mothers would have been least likely to attend antenatal appointments, which is where many mothers were recruited to the study.

For peer review only - http://bmjopen.bmj.com/site/about/guidelines.xhtml

Conclusions

Data linkage provides a means of identifying children with social services contact in cohort studies and of increasing their inclusion in research. The poor educational outcomes of the ALSPAC adolescents with social care records did not appear to be substantially explained by early-life exposures prior to contact with children's social care services, suggesting there may be scope for later interventions to make a difference.

For peer review only

For peer review only - http://bmjopen.bmj.com/site/about/guidelines.xhtml

Acknowledgments

We are extremely grateful to all the families who took part in this study, the midwives for their help in recruiting them, and the whole ALSPAC team, which includes interviewers, computer and laboratory technicians, clerical workers, research scientists, volunteers, managers, receptionists and nurses.

Conflicts of interest

JM is a foster carer. AT, AB and DW have no conflicts of interest to declare.

Funding

Core support for ALSPAC is provided by the UK Medical Research Council and Wellcome (Grant reference: 102215/2/13/2) and the University of Bristol. The Wellcome Trust (WT086118) funded ALSPACs linkage infrastructure through the Project to Enhance ALSPAC through Record Linkage (PEARL). This work was supported by the Elizabeth Blackwell Institute for Health Research, University of Bristol and the Wellcome Trust Institutional Strategic Support Fund (Grant number: 105612/Z/14/Z).

Contributor Statement

JM and AB conceived the study, and AT and JM developed the research question. AT conducted the analyses, interpreted the data and drafted the manuscript. JM and DW helped interpret the data and critically revised the paper. AB critically revised the paper. All authors have read and approved this final version.

Data Sharing Statement

The ALSPAC data management plan (available here: http://www.bristol.ac.uk/alspac/researchers/data-access/ documents/alspac-datamanagement-

For peer review only

For peer review only - http://bmjopen.bmj.com/site/about/guidelines.xhtml

plan.pdf) describes in detail the policy regarding data sharing, which is through a system of managed open access.

For peer review only

For peer review only - http://bmjopen.bmj.com/site/about/guidelines.xhtml

REFERENCES

1. Department for Education, National Statistics. Outcomes for Children Looked After by Local Authorities in England, as at 31 March 2016 (SRF 12/2017). 2017 [Available from: https://www.gov.uk/government/uploads/system/uploads/attachment_data/file/602087/SFR12_2017_Text.pdf.
2. Ford T, Vostanis P, Meltzer H, Goodman R. Psychiatric disorder among British children looked after by local authorities: comparison with children living in private households. The British Journal of Psychiatry. 2007;190(4):319-25.
3. Oswald SH, Heil K, Goldbeck L. History of Maltreatment and Mental Health Problems in Foster Children: A Review of the Literature. Journal of Pediatric Psychology. 2010;35(5):462-72.
4. Ward J, Henderson Z, Pearson G. One problem among many: drug use among care leavers in transition to independent living 2003 [Available from: http://www.drugsandalcohol.ie/5584/1/Home_Office_Research_Study_260_One_problem_among_many.pdf.
5. Viner RM, Taylor B. Adult Health and Social Outcomes of Children Who Have Been in Public Care: Population-Based Study. Pediatrics. 2005;115(4):894-9.
6. Botchway SK, Quigley MA, Gray R. Pregnancy-associated outcomes in women who spent some of their childhood looked after by local authorities: findings from the UK Millennium Cohort Study. BMJ Open. 2014;4(12).
7. Bellis MA, Lowey H, Leckenby N, Hughes K, Harrison D. Adverse childhood experiences: retrospective study to determine their impact on adult health behaviours and health outcomes in a UK population. Journal of Public Health. 2014;36(1):81-91.
8. Hughes K, Lowey H, Quigg Z, Bellis MA. Relationships between adverse childhood experiences and adult mental well-being: results from an English national household survey. BMC Public Health. 2016;16(1):222.
9. Plant DT, Jones FW, Pariante CM, Pawlby S. Association between maternal childhood trauma and offspring childhood psychopathology: mediation analysis from the ALSPAC cohort. The British Journal of Psychiatry. 2017.
10. Teyhan A, Wijedasa D, Macleod J. Adult psychosocial outcomes of men and women who were looked-after or adopted as children: prospective observational study. BMJ Open. 2018.
11. Children's commissioner for England. On measuring the number of vulnerable children in England 2017 [Available from: https://www.childrenscommissioner.gov.uk/wp-content/uploads/2017/07/CCO-On-vulnerability-Overveiw-2.pdf.
12. Mezey G, Robinson F, Campbell R, Gillard S, Macdonald G, Meyer D, et al. Challenges to undertaking randomised trials with looked after children in social care settings. Trials. 2015;16(1):206.
13. Department for Education. Children in need census 2017 to 2018. Guide for local authorities - version 1.2 2018 [Available from: https://assets.publishing.service.gov.uk/government/uploads/system/uploads/attachment_data/file/695113/CIN17-18_Guide_v1.2-web_version.pdf.
14. Department for Education. Characteristics of children in need: 2016 to 2017 England (SFR 61/2017)2017 Accessed February 2018. Available from: https://www.gov.uk/government/uploads/system/uploads/attachment_data/file/656395/SFR61-2017_Main_text.pdf.
15. Department for Education, National Statistics. Children looked after in England (including adoption), year ending 31 March 2017 (SFR 20/2017)2017 Accessed February 2018. Available from: https://www.gov.uk/government/uploads/system/uploads/attachment_data/file/664995/SFR50_2017-Children_looked_after_in_England.pdf.
16. Fletcher J, Strand S, Thomas S. The Educational Progress of Looked After Children in England.

For peer review only - http://bmjopen.bmj.com/site/about/guidelines.xhtml

Technical Report 1: Secondary School Progress and Attainment 2015 [Available from: http://reescentre.education.ox.ac.uk/wordpress/wp-content/uploads/2015/11/EducationalProgressLookedAfterChildrenTechnical-Report-1_Nov2015.pdf.
17. Sebba J, Berridge D, Luke N, Fletcher J, Bell K, Strand S, et al. The Educational Progress of Looked After Children in England: Linking Care and Educational Data 2015 [Available from: http://reescentre.education.ox.ac.uk/wordpress/wp-content/uploads/2015/11/EducationalProgressLookedAfterChildrenOverviewReport_Nov2015.pdf.
18. Berridge D. Educating young people in care: What have we learned? Children and Youth Services Review. 2012;34(6):1171-5.
19. Boyd A, Golding J, Macleod J, Lawlor DA, Fraser A, Henderson J, et al. Cohort Profile: The 'Children of the 90s'—the index offspring of the Avon Longitudinal Study of Parents and Children. International Journal of Epidemiology. 2012.
20. Fraser A, Macdonald-Wallis C, Tilling K, Boyd A, Golding J, Davey Smith G, et al. Cohort Profile: The Avon Longitudinal Study of Parents and Children: ALSPAC mothers cohort. International Journal of Epidemiology. 2013;42(1):97-110.
21. ALSPAC. Data Dictionary [Available from: http://bristol.ac.uk/alspac/researchers/data-access/data-dictionary/.
22. Boyd A, Tilling K, Cornish R, Davies A, Humphries K, Macleod J. Professionally designed information materials and telephone reminders improved consent response rates: evidence from an RCT nested within a cohort study. Journal of Clinical Epidemiology. 2015;68(8):877-87.
23. Mc Grath-Lone L, Harron K, Dearden L, Nasim B, Gilbert R. Data Resource Profile: Children Looked After Return (CLA). International Journal of Epidemiology. 2016;45(3):716-7f.
24. Emmott E, Jay M, Woodman J. Cohort Profile: Children in Need Census (CIN) Records of Vulnerable Children in England (pre-print). BMJ Open. 2018.
25. Department for Education. The National Pupil Database: User Guide 2015 [Available from: www.gov.uk/government/uploads/system/uploads/attachment_data/file/472700/NPD_user_guide.pdf.
26. National Pupil Database in England Wiki. KS4 2016 [Available from: https://nationalpupildatabase.wikispaces.com/KS4.
27. Thorpe K. A study of the use of the Edinburgh postnatal depression scale with parent groups outside the postpartum period. Journal of Reproductive and Infant Psychology. 1993;11(2):119-25.
28. GOV.UK. Apply for free school meals 2018 [Available from: https://www.gov.uk/apply-free-school-meals.
29. Department for Communities and Local Government. The English Indices of Deprivation 2015 [Available from: https://www.gov.uk/government/uploads/system/uploads/attachment_data/file/465791/English_Indices_of_Deprivation_2015_-_Statistical_Release.pdf.
30. Hirsch D. Experiences of poverty and educational disadvantage 2007 [Available from: https://www.jrf.org.uk/report/experiences-poverty-and-educational-disadvantage.
31. Goodman A, Gregg P. Poorer children's educational attainment: how important are attitudes and behaviour? 2010 [Available from: https://www.jrf.org.uk/report/poorer-children%E2%80%99s-educational-attainment-how-important-are-attitudes-and-behaviour.
32. Lo A, Roben CKP, Maier C, Fabian K, Shauffer C, Dozier M. "I want to be there when he graduates:" Foster parents show higher levels of commitment than group care providers. Children and Youth Services Review. 2015;51:95-100.
33. Luke N, Sinclair I, O'Higgins A. The Educational Progress of Looked After Children in England. Technical report 2: Relating Care to Educational Attainment and Progress 2015 [Available from: https://weblearn.ox.ac.uk/access/content/group/5cacf183-ce54-47f0-9f17-d4b0fe1cf292/EducationalProgressLookedAfterChildrenTechnical%20Report%202_Nov2015.pdf.

For peer review only - http://bmjopen.bmj.com/site/about/guidelines.xhtml

34. Hutchings HA, Evans A, Barnes P, Demmler J, Heaven M, Hyatt MA, et al. Do children who move home and school frequently have poorer educational outcomes in their early years at school? An anonymised cohort study. PloS one. 2013;8(8):e70601.
35. Strand S, Demie F. Pupil mobility, attainment and progress in secondary school. Educational Studies. 2007;33(3):313-31.

For peer review only

For peer review only - http://bmjopen.bmj.com/site/about/guidelines.xhtml

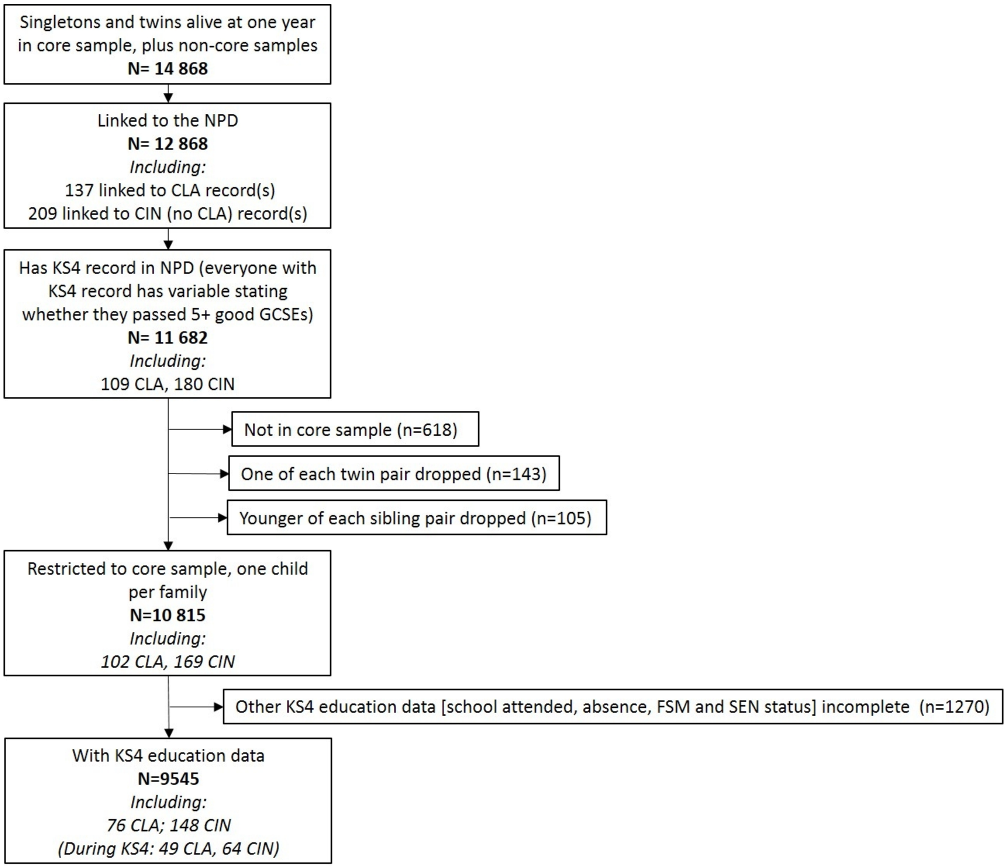

Figure 1 - Flow Chart of Sample

218x189mm (150 x 150 DPI)

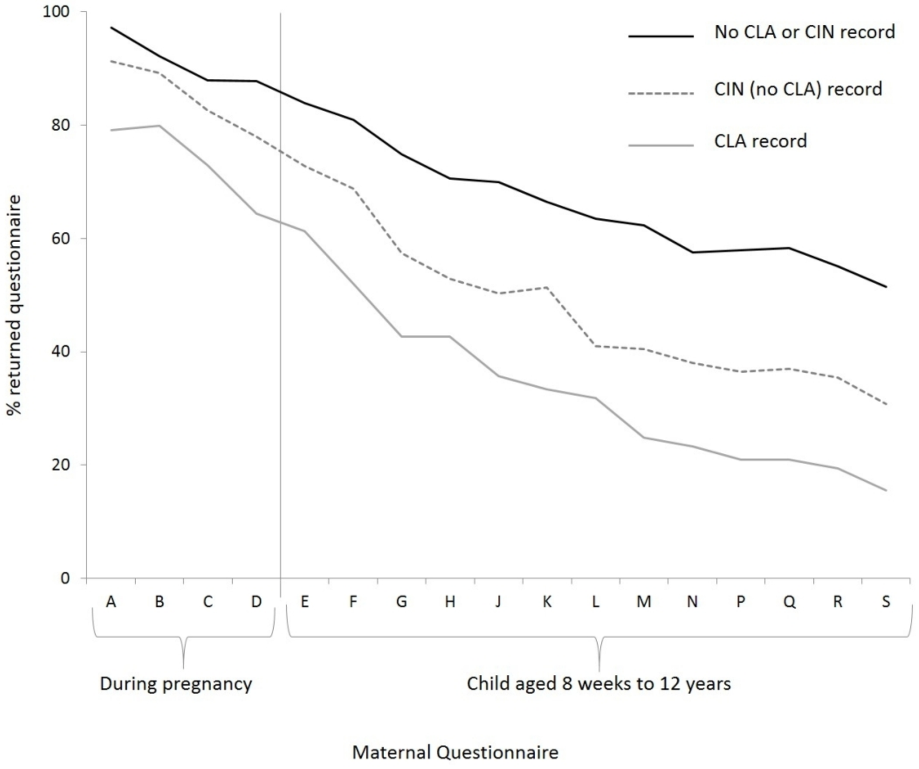

Notes on figure:

Sample restricted to mothers in the core sample, whose child has been linked to the National Pupil Database.

Mothers may not have completed every question within each questionnaire.

Figure 2 - Maternal questionnaire response rates by child social care status

231x223mm (150 x 150 DPI)

Supplementary Text - Additional detail on covariates

- **Early-life, maternal and family characteristics**

The following maternal, family and socioeconomic measures were reported by the mothers during their pregnancy with the study child via postal questionnaires. The categories for each variable are given in parenthesis following the variable name.

Highest educational qualification (university degree/A level; O level; vocational/none)

Financial Difficulties (quartiles of score with range 0–40, where 0 is no financial difficulties)

Housing tenure (owned/mortgaged; private rent; council rent; other)

Partner status (husband; live with partner; do not live with partner/no partner)

Smoking during pregnancy (no; yes)

Low social support - measured by response to 10 items with a low score defined as being in the bottom 10% (no; yes).

Maternal depression - measured by the Edinburgh Postnatal Depression Scale. Although this measure was originally designed for use with postnatal women, none of the 10 items is specific to this period and it has been validated for use at other times; it was chosen as it does not contain somatic items that could confound normal symptoms in pregnancy with depression (27). Quartiles were derived.

- **Proxy measures of socio-economic position when child aged 16**

The following measures were obtained from the National Pupil Database.

For peer review only

Child in receipt of free school meals (FSM) - eligibility is based on low parental income (28) (no; yes)

Child's residential neighbourhood deprivation - measured by the Income Deprivation Affecting Children Index (IDACI) (29), which gives the proportion of children (<0-15yrs) in a neighbourhood (lower super output area, average population 1500) who live in a low income family (<10%; 10 to <20%; 20 to <30%; 40%+).

For peer review only

Supplementary (online only) Tables

Table A: Summary of CLA and CIN data availability by year and period of birth

Table B: Summary of early-life (maternal and family) variables before and after multiple imputation

Table C: Summary of CIN data for ALSPAC participants linked to a post-2008 CLA record

Table D: Summary of care data for ALSPAC participants linked to a post-2005 CLA record

Table E: Comparison of care characteristics between ALSPAC CLA participants and CLA individuals in general population (in ALSPAC area and in England) of same age.

Table F: Educational attainment, absence, and special educational needs by care status – no SEN

Table G: Educational attainment, absence, and special educational needs by care status – no disability

For peer review only

Table A: Summary of CLA and CIN data availability by year and period of birth

	Expected school year in March of each year[1]					
Period of birth	2006[2]	2007[2]	2008[2]	2009[2,3]	2010[2,3]	2011[2,3]
April 1991 to August 1991	Year 10	**Year 11[4]**	Year 12	Year 13		
September 1991 to August 1992	Year 9	Year 10	**Year 11[4]**	Year 12	Year 13	
September 1992 to January 1993	Year 8	Year 9	Year 10	**Year 11[4]**	Year 12	Year 13

[1] The school year in England runs from September to July. In contrast, the CLA and CIN data collection year runs from April to March. For example, the 2006 CLA dataset would cover the period from April 2005 to March 2006; the oldest ALSPAC participants would have been in Year 10 in March 2006, and the youngest in Year 8.

[2] CLA data linked to NPD available

[3] CIN data linked to NPD available

[4] GCSE exams are taken in May/June of Year 11.

Determining who had CIN record during KS4:

For the CIN data, the youngest cohort participants were in Year 11 at the time of the earliest CIN data collection and they were coded as being ‘in need during KS4’ if they had a CIN record. However, the majority of the cohort were already in Year 12 or 13 at this time, and so we calculated the age they had been referred, and identified those who had been referred before they sat their KS4 exams (age on the 1st June of the year they were in Year 11 was used as a proxy for age that exams were taken).

For peer review only

Table B: Summary of early-life (maternal and family) variables before and after multiple imputation

Variables with missing data in study sample[1]	% missing	Categories[2]	Study sample [N=9545]	
			Before imputation [N specified for each variable individually]	Imputed [N=9545]
**Variables reported by mother during pregnancy**				
Relationship status	5.2		N=9048	
		Married (%)	75.7 (74.8-76.6)	74.7 (73.8-75.6)
		Resident partner (%)	16.1 (15.3-16.8)	16.5 (15.8-17.3)
		Non-resident/no partner (%)	8.2 (7.7-8.8)	8.7 (8.1-9.3)
Highest maternal education	9.7		N=8623	
		A Level or degree (%)	31.7 (30.8-32.7)	30.5 (29.5-31.4)
		O Level	36.8 (35.8-37.9)	36.5 (35.5-37.5)
		Vocational/none (%)	31.4 (30.4-32.4)	33.0 (32.0-34.0)
Financial difficulties	12.1		N=8387	
		Q1 (none) (%)	34.9 (33.9-35.9)	32.7 (32.7-34.7)
		Q4 (high) (%)	20.4 (19.5-21.3)	21.5 (20.6-22.4)
Housing tenure	5.7		N=9003	
		Owned/mortgaged (%)	74.6 (73.6-75.4)	73.4 (72.5-74.3)
Maternal smoking	5.0		N=9068	
		Yes (%)	26.2 (25.3-27.1)	26.9 (26.0-27.8)
	13.1		N=8294	
Depression score		Highest quartile (%)	22.7 (21.8-23.6)	23.5 (22.6-24.4)
Low social support	16.8		N=7942	
		Yes (%)	9.3 (8.7-10.0)	10.5 (9.7-11.2)
**Educational attainment from NPD**				
Capped percentage point score[3]	0.9	Mean	68.8 (68.4-69.2)	68.5 (68.1-68.9)

[1] The following variables had no missing data in study sample: child age, sex, maternal age at delivery, attainment of 5+ good GCSEs, persistent absence, SEN status, school attended.

[2] For brevity, not all categories are presented for each variable.

[3] The binary attainment variable (5+ good GCSEs) was complete for all those with a KS4 NPD record, but a small number (n=82) had missing capped point score data.

For peer review only

Table C: Summary of CIN data for ALSPAC participants linked to a post-2008 CIN record (but no CLA record)

Variable		CIN (no CLA) record N=209
Age (yrs) at referral date	Median (range)	16.7 (2.5-18.1)
Primary need status	Abuse or neglect (%, 95% CI)	22.0 (16.9-28.2)
	Child disability/illness (%, 95% CI)	23.4 (18.2-29.7)
	Parental illness/disability (%, 95% CI)	[n<5]
	Family in acute stress (%, 95% CI)	21.5 (16.4-27.7)
	Family dysfunction (%, 95% CI)	18.2 (13.5-24.1)
	Socially unacceptable behaviour (%, 95% CI)	[n<5]
	Low income (%, 95% CI)	[n<5]
	Absent parenting (%, 95% CI)	[n<5]
	Cases other than CIN (%, 95% CI)	[n<5]
	Not stated (%, 95% CI)	10.0 (6.6-15.0)
Child ever coded as having disability	Yes (%, 95% CI)	22.0 (16.9-28.2)

For peer review only

Table D: Summary of care data for ALSPAC participants linked to a post-2005 CLA record

		Linked to CLA record		
Variable		Overall<br><br>n=137	Eligible for one-third sample[1]<br>n=47	Ineligible for one-third sample[2]<br>n=90
Total number of periods of care[3]	Median, range	1 (1-13)	1 (1-8)	1 (1-13)
Total number of episodes of care[3]	Median, range	3 (1-28)	3 (1-24)	3 (1-28)
Age (yrs) at start of first period of care[3]	Median, range	13.2 (0-17.8)	11.5 (0-17.6)	13.7 (0.1-17.8)
Age (yrs) at end of last episode of care[3]	Median, range	17.7 (13.1-18.0)	18.0 (13.1-18.0)	17.1 (13.2-18.0)
		N=134	N=45	N=89
Total duration (days) in care [3,4]	Median, range	906 (1-5736)	1394 (2-5498)	604 (1-5736)
Primary need category at start of first period of care[3,5]	Abuse or neglect (%, 95% CI)	30.7 (23.4-39.0)	34.4 (21.6-49.1)	28.9 (20.3-39.3)
	Child disability (%, 95% CI)	16.1 (10.8-23.3)	19.1 (10.0-33.5)	14.4 (8.5-23.5)
	Parental illness/disability (%, 95% CI)[6]	4.4 (2.0-9.5)	/	/
	Family in acute stress (%, 95% CI)	18.2 (12.6-25.7)	17.0 (8.5-31.1)	18.9 (12.0-28.5)
	Family dysfunction (%, 95% CI)	17.5 (12.0-24.9)	25.5 (14.8-40.4)	13.3 (7.6-22.2)
	Socially unacceptable behaviour (%, 95% CI)[6]	8.8 (5.0-14.9)	/	/
	Absent parenting (%, 95% CI)[6]	4.4 (2.0-9.5)	/	/
Placement type for last episode of care	Foster care (%, 95% CI)	62.8 (54.3-70.5)	57.4 (42.6-71.1)	65.6 (55.0-74.8)
	Children's/residential/care home or school (%, 95% CI)	18.2 (12.6-25.7)	23.4 (13.2-38.1)	15.6 (9.3-24.8)
	Other (%, 95% CI)	19.0 (13.2-26.5)	19.1 (10.0-33.5)	18.9 (12.0-28.5)
Reason for last episode of care ending	Returned home to parents or relatives (%, 95% CI)	36.5 (28.8-45.0)	27.7 (16.5-42.6)	41.1 (31.3-51.7)
	Moved to independent living (%, 95% CI)	16.8 (11.4-24.1)	19.1 (10.0-33.4)	15.6 (9.3-24.8)
	Residential care funded by adult social services (%, 95% CI)	13.1 (8.3-20.0)	21.3 (11.6-35.8)	8.9 (4.4-17.0)
	Other (%, 95% CI)	32.8 (25.4-41.2)	31.9 (19.9-47.0)	34.4 (25.2-45.0)

[1]Individuals eligible for the one-third sample will have CLA records in the period 1998-2003 if they were looked-after during this time.
[2] No CLA data were collected from 2008-2003 for those ineligible for the one-third sample; therefore their looked-after status during this period is unknown.
[3] Measures relate to the data available from linkage only: these should be complete for those eligible for the one-third sample, but will not be for those in the ineligible sample who were looked-after during 1998-2003.
[4]The sum of the duration of all episodes of care, which may or may not have been consecutive.
[5]Refers to primary need at the start of the first period of care for which we have a record.
[6]Percentages not shown by one-third sample status for these need categories to prevent derivation of small cell counts (n<5).

Table E: Comparison of care characteristics between ALSPAC participants with CLA records and individuals with CLA records in general population of same age (in ALSPAC area and in England).

		Born 1991 or 1992 with CLA record in England[1]<br><br>n=43938	Born 1991 or 1992 with CLA record in ALSPAC area[1,2]<br><br>n=713	ALSPAC participants with CLA record<br><br>n=137
Total number of periods of care [3]	Median, range	1 (1-516)	1 (1-46)	1 (1-13)
Total number of episodes of care[3]	Median, range	2 (1-517)	2 (1-49)	3 (1-28)
Age (yrs) at start of first period of care[3]	Median, range	9.7 (0-18)	7.6 (0-18)	13.2 (0-17.8)
Age (yrs) at end of last episode of care[3]	Median, range	15.5 (0-19.9)	14.2 (0-18.2)	17.7 (13.1-18.0)
Last period of care ended <12yrs	Yes (%, 95% CI)	35.6 (35.2-36.1)	41.9 (38.4-45.6)	0
		n=43554	n=707	n=134
Total duration (days) in care [3,4]	Median, range	461 (1-6575)	427 (1-6069)	906 (1-5736)
		n=30250	n=453	n=137
Primary need category at start of first period of care[3,5]	Abuse or neglect (%, 95% CI)	35.3 (34.8-35.9)	26.3 (22.4-30.5)	30.7 (23.4-39.0)
	Child disability (%, 95% CI)	12.7 (12.4-13.1)	16.1 (13.0-19.8)	16.1 (10.8-23.3)
	Parental illness/disability (%, 95% CI)	3.9 (3.7-4.1)	5.1 (3.4-7.5)	4.4 (2.0-9.5)
	Family in acute stress (%, 95% CI)	10.9 (10.5-11.2)	18.5 (15.2-22.4)	18.2 (12.6-25.7)
	Family dysfunction (%, 95% CI)	12.4 (12.0-12.8)	20.5 (17.0-24.5)	17.5 (12.0-24.9)
	Socially unacceptable behaviour (%, 95% CI)	6.3 (6.1-6.6)	6.2 (4.3-8.8)	8.8 (5.0-14.9)
	Absent parenting (%, 95% CI)	18.1 (17.7-18.6)	7.3 (5.2-10.1)	4.4 (2.0-9.5)
		N=43602	N=712	N=137
Placement type for last episode of care	Foster care	32.8 (32.4-33.3)	42.0 (38.4-45.7)	62.8 (54.3-70.5)
	Children's/residential/care home/school	14.1 (13.7-14.4)	9.4 (7.5-11.8)	18.2 (12.6-25.7)
	Other	53.1 (52.6-53.6)	49.0 (44.9-52.3)	19.0 (13.2-26.5)
		N=39,647	N=644	N=137
Reason for last episode of care ending	Returned home to parents or relatives	21.9 (21.5-22.3)	24.8 (21.7-28.3)	36.5 (28.8-45.0)
	Moved to independent living	17.7 (17.4-18.1)	14.8 (12.2-17.7)	16.8 (11.4-24.1)
	Residential care funded by adult social services	4.8 (4.5-5.0)	7.6 (5.8-9.9)	13.1 (8.3-20.0)

[1]Excludes the 137 individuals identified as being in ALSPAC

[2]Includes only those in the care of one of the following local authorities: City of Bristol; Bath and North East Somerset; South Gloucestershire; North Somerset.

For peer review only

Table F: Educational attainment, absence, and special educational needs by care status excluding those with SEN (n=8145)

Outcome	Care status during KS4	Model 1[1] (Age and Sex)	Model 2[2] (KS4 variables)	Model 3[3] (Early-life variables)	Model 4[4] (Fully adjusted)
		OR (95% CI)	OR (95% CI)	OR (95% CI)	OR (95% CI)
5 A*-C GCSEs inc. Eng. & Maths	Not CIN or CLA	Ref	Ref	Ref	Ref
	CIN (not CLA)	0.04 (0.01-0.36)	0.08 (0.01-0.65)	0.08 (0.01-0.66)	0.11 (0.01-0.99)
	CLA	0.26 (0.08-0.77)	0.31 (0.10-0.98)	0.35 (0.11-1.11)	0.39 (0.12-1.31)
		Coeff (95% CI)	Coeff (95% CI)	Coeff (95% CI)	Coeff (95% CI)
Capped percentage score	Not CIN or CLA	Ref	Ref	Ref	Ref
	CIN (not CLA)	-24.2 (-31.9 to -16.6)	-10.7 (-17.7 to -3.7)	-18.1 (-25.2 to -11.1)	-8.8 (-15.4 to -2.1)
	CLA	-26.7 (-33.3 to -20.1)	-21.1 (-27.1 to -15.1)	-22.7 (-28.8 to -16.6)	-18.6 (-24.3 to -12.9)

[1]Adjusted for child age and sex
[2]Adjusted for child age and sex, plus KS4 time-point variables (persistent school absence, in receipt of free school meals, school mobility, IDACI of residential neighbourhood)
[3]Adjusted for child age and sex, plus early-life[maternal and SEP] variables (maternal age at delivery, education, partner status, housing tenure, financial difficulties, smoking, depression, social support)
[4]Adjusted for child age and sex, plus KS4 and early life variables

Table G: Educational attainment, absence, and special educational needs by care status excluding those with a disability (n=9506)

Outcome	Care status during KS4	Model 1[1] (Age and Sex)	Model 2[2] (KS4 variables)	Model 3[3] (Early-life variables)	Model 4[4] (Fully adjusted)
		OR (95% CI)	OR (95% CI)	OR (95% CI)	OR (95% CI)
5 A*-C GCSEs inc. Eng. & Maths	Not CIN or CLA	Ref	Ref	Ref	Ref
	CIN (not CLA)	0.07 (0.02-0.25)	0.12 (0.03-0.43)	0.12 (0.03-0.44)	0.18 (0.05-0.64)
	CLA	0.17 (0.07-0.43)	0.19 (0.07-0.49)	0.28 (0.11-0.74)	0.29 (0.11-0.76)
		Coeff (95% CI)	Coeff (95% CI)	Coeff (95% CI)	Coeff (95% CI)
Capped percentage score	Not CIN or CLA	Ref	Ref	Ref	Ref
	CIN (not CLA)	-25.7 (-31.7 to -19.7)	-13.0 (-18.5 to -7.4)	-19.2 (-24.8 to -13.6)	-11.0 (-16.2 to -5.7)
	CLA	-28.6 (-34.3 to -23.0)	-24.6 (-29.8 to -19.5)	-22.7 (-27.9 to -17.5)	-20.7 (-25.6 to -15.8)

[1]Adjusted for child age and sex
[2]Adjusted for child age and sex, plus KS4 time-point variables (persistent school absence, in receipt of free school meals, school mobility, IDACI of residential neighbourhood)
[3]Adjusted for child age and sex, plus early-life[maternal and SEP] variables (maternal age at delivery, education, partner status, housing tenure, financial difficulties, smoking, depression, social support)
[4]Adjusted for child age and sex, plus KS4 and early life variables

For peer review only

Supplementary Figure 3: Partner, child and teacher questionnaire response rates by child social care status

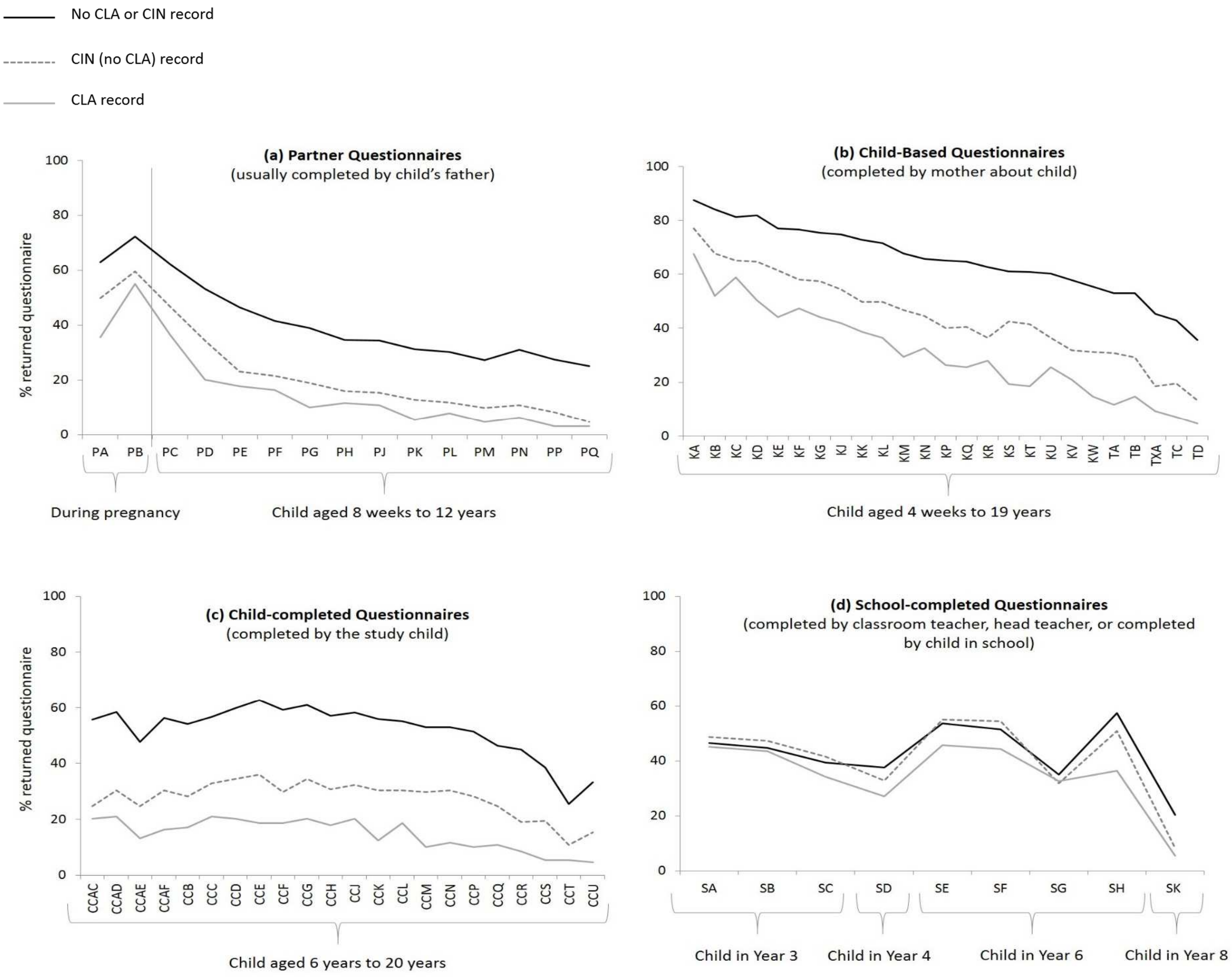

BMJ Open

Early-life adversity, contact with children's social care services, and educational outcomes at age 16 years: UK birth cohort study with linkage to national administrative records

Journal:	*BMJ Open*
Manuscript ID	bmjopen-2019-030213.R1
Article Type:	Original research
Date Submitted by the Author:	15-Aug-2019
Complete List of Authors:	Teyhan, Alison; University of Bristol, Bristol Medical School, Population Health Sciences<br>Boyd, Andy; University of Bristol, Bristol Medical School, Population Health Sciences<br>Wijedasa, Dinithi; University of Bristol, Hadley Centre for Adoption and Foster Care Studies, School for Policy Studies<br>Macleod, John; University of Bristol, Bristol Medical School, Population Health Sciences
**Primary Subject Heading**:	Epidemiology
Secondary Subject Heading:	Epidemiology, Research methods
Keywords:	ALSPAC, record linkage, education, social care, looked-after, adolescence

SCHOLARONE™
Manuscripts

Early-life adversity, contact with children's social care services, and educational outcomes at age 16 years: UK birth cohort study with linkage to national administrative records

Alison Teyhan*[a], Andy Boyd[a], Dinithi Wijedasa[b], John Macleod[a]

*Corresponding author

Email: alison.teyhan@bristol.ac.uk

Phone: +44 (0)117 331 0058

[a] Population Health Sciences, Bristol Medical School, University of Bristol, Oakfield House, Oakfield Grove, Bristol, BS8 2BN, UK

[b] Hadley Centre for Adoption and Foster Care Studies, School for Policy Studies, University of Bristol, 8 Priory Road, Bristol, BS8 1TZ, UK

For peer review only

For peer review only - http://bmjopen.bmj.com/site/about/guidelines.xhtml

UOB Confidential & Sensitive

ABSTRACT (248 words)

Objectives: To use record linkage of birth cohort and administrative data to study educational outcomes of children who are looked-after (in public care) and in need (social services involvement), and examine the role of early life factors.

Setting, Design: Prospective observational study of children from the Avon Longitudinal Study of Parents and Children (ALSPAC), which recruited pregnant women in and around Bristol, UK in the early 1990s. ALSPAC was linked to the annual Children Looked-After (CLA) Data Return and Children In Need (CIN) Census. Educational outcomes at 16 years were obtained through linkage to the National Pupil Database. These included passing 5+ good GCSEs (grades A*-C, including English and Maths). Covariates included early-life adversity and social position.

Participants: 9545 children from ALSPAC who had complete education and covariate data were included in the main educational outcomes analyses.

Results: Overall, of the 12,868 ALSPAC participants linked to NPD data, 137 had a CLA record and a further 209 a CIN record during adolescence. These children were more disadvantaged than their peers and had little active study participation beyond infancy. In the main educational outcomes analyses, achievement of 5+ good GCSEs was low in the CLA (OR 0.14, 95% CI 0.05-0.35) and CIN (0.11, 0.05-0.27) groups relative to their peers. Measured early-life factors explained little of this difference.

Conclusions: Data linkage enabled the study of educational outcomes in children with social services contact. These children had substantially worse educational outcomes relative to their peers, for reasons likely to be multifactorial.

Key Words: ALSPAC; record linkage; education; social care; looked-after; adolescence

For peer review only - http://bmjopen.bmj.com/site/about/guidelines.xhtml

UOB Confidential & Sensitive

Article Summary - Strengths and Limitations

- We link a population-based birth cohort study (ALSPAC) to social care and educational records, and demonstrate that record linkage offers a means to identify vulnerable children in a cohort and increase their inclusion in research.
- The children in ALSPAC who had been looked-after (in public care) were broadly representative in terms of their care characteristics of children nationally of the same age who had been looked-after.
- We were only able to identify children who had been in care or in need during adolescence.
- Cohort data availability for children with social care records in adolescence was low beyond infancy.

For peer review only

For peer review only - http://bmjopen.bmj.com/site/about/guidelines.xhtml

UOB Confidential & Sensitive

INTRODUCTION

Children with social services contact, including those in public care, are at higher risk of poor outcomes than their peers, including low educational attainment, substance abuse, and mental illness(1-10). The extent to which this reflects early-life adversity prior to contact with services as opposed to later influences is unclear. Outcomes mainly resulting from early adversity may be less amenable to change through social care interventions, requiring alternative prevention strategies. These children are challenging to study using traditional research methods. A recent Children's Commissioner for England report highlights that vulnerable children are 'absent or poorly measured in national studies'(11), and children's social care is a difficult area in which to conduct randomised controlled trials(12). Further, those who experience extreme adversity are likely under-represented in birth cohort studies due to low recruitment and high attrition, and identification of vulnerable children is challenging due to reliance on parental-report.

Children with social services contact in England do however have high levels of administrative data. The term 'in need' refers to children who have been referred to and assessed by social services and found to be 'unlikely to achieve or maintain a reasonable level of health or development, or whose health and development is likely to be significantly or further impaired, without the provision of services; or a child who is disabled'(13). Almost 390,000 children are currently classified as 'in need'(14). Some children in need may enter the public care system and become a 'looked-after' child. Presently over 72,000 children are looked-after(15), with the majority placed with foster carers(1).

While routine statistics using social care data can highlight poor outcomes, e.g. low average educational attainment, they lack information on early-life and family characteristics(1, 16, 17). These types of data are readily available in birth cohort studies. Linking cohort data to

For peer review only - http://bmjopen.bmj.com/site/about/guidelines.xhtml

UOB Confidential & Sensitive

social care records could therefore provide a means of identifying children in need and looked-after without reliance on parental-report. Further, using additional linked data to measure outcomes potentially enables the child's inclusion in analyses even if their family have stopped actively participating in the cohort study.

We use record linkage to a birth cohort to examine the effect of being in need or looked-after in adolescence on educational outcomes at age 16 years: the low attainment of many in need and looked-after children at this age is a concern as it can compound their disadvantaged childhoods to limit future education, employment, and general life chances(18).

METHODS

Data

Avon Longitudinal Study of Parents and Children (ALSPAC)

Pregnant women living in and around the city of Bristol, UK with expected date of delivery April 1991 to December 1992 were eligible to participate in ALSPAC. There were 14,541 pregnancies enrolled, resulting in 13,988 children alive at one year, including 13,972 singletons and twins. This 'core sample' was later bolstered by further eligible children: an additional 713 from age 7-18yrs, and to date 183 since age 18yrs. The mothers, their partners, and the study children are studied via questionnaires and clinic visits. Teachers also completed questionnaires on the children. Further details are provided in cohort profiles(19, 20) and searchable data-dictionary(21). For the main analyses on educational outcomes, the sample was restricted to: core, one child per family, with education data (n=9545, Figure 1).

Ethical approval for ALSPAC was obtained from the ALSPAC Ethics and Law Committee and Local Research Ethics Committees (www.bristol.ac.uk/alspac/researchers/research-ethics/). When study children reached age 18, they were sent 'fair processing' materials

For peer review only - http://bmjopen.bmj.com/site/about/guidelines.xhtml

UOB Confidential & Sensitive

which described ALSPAC's intended use of their health and administrative records, and gave a clear means to object(22). Education data were not extracted for participants who objected, or who were not sent fair processing materials.

Linkage data

Data on children who are looked-after, or have been referred as a child in need, are collected annually via the Children Looked-After (CLA) Data Return(23) and the Children in Need (CIN) Census(24). The CIN Census covers all children referred to children's social services even if no further action is taken. The CLA Return and the CIN Census have been linked to the National Pupil Database (NPD), a repository of education data for schools in England(25), since their 2005/06 and 2008/09 data collections respectively. ALSPAC has an established link to the NPD, and thus to any post-2005 CLA or post-2008 CIN record for participants in the NPD. Earlier CLA records were also obtained for those with a post-2005 record. However, CLA data collection was only on a random one-third sample of looked-after children from 1998-2003, meaning no records exist for many looked-after children in this period(23). Insufficient identifiers exist within the CLA dataset to enable linkage of ALSPAC to pre-2005 CLA records for those without a post-2005 record.

We also obtained CLA records for all individuals in the CLA Return of a similar age (born January 1991-December 1992) to form two comparison groups: (1) ever looked-after in England (n=43,938); (2) ever looked-after in the four local authorities that approximate the ALSPAC recruitment area (Bristol City; South Gloucestershire; North Somerset; Bath and North East Somerset) (n=713).

Measures

Educational outcomes

For peer review only

UOB Confidential & Sensitive

Pupils in England study General Certificate of Education (GCSE) courses during Key Stage 4 (KS4) of their education (Years 10 and 11, aged 14-16yrs) and take GCSE exams at the end of Year 11. The oldest ALSPAC children sat their GCSE exams in 2007, the youngest in 2009. Our main outcomes were two measures of attainment. First, a binary measure: achievement of 5+ good GCSEs (grades A*-C, including English and Maths). Second, a continuous measure: capped point score, expressed as a percentage of the maximum possible capped point score (based on the eight best grades obtained, with each grade assigned a numerical value)(26). Secondary educational outcomes included: persistent absence (≥10% of half days); special educational needs (SEN) status (see Supplementary Text for definitions of the different SEN categories); and school mobility (whether child joined school during KS4).

Contact with children's social care services

Contact with children's social care services (referred to as 'social care status' hereafter) was summarised in two variables. The first specified whether a child had any post-2005 CLA record(s) or post-2008 CIN record(s) (i.e. was looked-after or referred to social care services at any time for which we have linked social care data). The second summarised social care status during KS4 only. This restriction was necessary for the educational outcomes analyses to ensure our exposure preceded our outcome, plus these are the only two school years with CLA data coverage for all children in our sample (Supplementary Table A). By definition children who are looked-after are also in need but we use 'in need' to refer to children with a CIN but not a CLA record. The reference group comprised children with a KS4 record in the NPD who had no linked social care record.

Variables related to being in care or in need were derived from the linked data as follows. CIN Census: category of need; age referred. CLA Return: category of need; age first period of

For peer review only - http://bmjopen.bmj.com/site/about/guidelines.xhtml

UOB Confidential & Sensitive

care (POC) started (POC is a period of time when child is continuously looked-after by the local authority); number of POC and episodes of care (a POC is comprised of 'episodes', each representing a period of being looked-after under the same legal status and in the same placement); placement type (foster; children's home/residential home/residential school; other [no further disaggregation possible due to small numbers]).

Covariates

These included child age and sex, plus measures related to family socio-economic position (SEP). Early-life exposures included maternal age at delivery, and measures reported by the mother during pregnancy: highest educational qualification; financial difficulties; housing tenure; partner status; smoking; alcohol intake; social support; and depressive symptoms(27). Later measures of SEP (during KS4) were obtained from the NPD: receipt of free school meals (FSM)(28); and child's residential neighbourhood deprivation measured by the Income Deprivation Affecting Children Index (IDACI)(29). More details in Supplementary Text.

Statistical analyses

Descriptive statistics were used to: summarise the social care data linked to ALSPAC children; compare the ALSPAC looked-after sample to the two non-ALSPAC looked-after comparison groups; compare child, maternal and SEP characteristics by social care status; describe questionnaire completion rates by social care status.

Associations between social care status and educational outcomes were examined using multilevel regression models (individual level 1, school level 2). Linear models were used for capped point score, logistic for attainment of 5+ good GCSEs. Associations were adjusted for age and sex (Model 1), then also for KS4 measures [FSM, neighbourhood deprivation, school mobility] (Model 2), or for early-life exposures (Model 3). We then adjusted for all KS4 and early-life variables (Model 4). Multiple imputation using chained equations was used to

For peer review only - http://bmjopen.bmj.com/site/about/guidelines.xhtml

UOB Confidential & Sensitive

For peer review only

impute missing data (supplementary Table B) for the educational outcomes analyses sample (n=9545). 100 datasets were imputed.

In sensitivity analyses, models were restricted to children with no SEN (n=8145) or no disability (n=9506). Social care status at any time was also considered. Finally, we described associations between social care characteristics (e.g. placement type, reason for being in need) and capped point score in those with CIN or CLA records: to maximise sample size, we included all those who had these records at any time and who had capped point score data.

Patient and Public Involvement

Patients, the public, and study participants were not directly involved in this study. Some ALSPAC participants are members of a committee which meets bi-monthly to provide insights and advice on general ALSPAC study design, methodology and acceptability for participants.

RESULTS

Children in ALSPAC with social care records

Of those with a post-2008 CIN (but no CLA) record (n=209) the most common needs at referral were child disability, abuse or neglect, and family in acute stress. Of those with a post-2005 CLA record (n=137), the most common primary need was abuse or neglect (Supplementary Tables C and D). Median total time in care was 2.6 years. Foster care was the most common placement type.

Comparison to non-ALSPAC looked-after children

The ALSPAC children with CLA records were generally similar to those of children born at the same time who were ever in care in England (comparison group 1) or in the area in and

For peer review only - http://bmjopen.bmj.com/site/about/guidelines.xhtml

UOB Confidential & Sensitive

For peer review only

around Bristol (comparison group 2) in terms of primary need (Supplementary Table E). Importantly, many of those who had ever had a CLA record in the two comparison groups (36% of group 1; 42% of group 2) had left care before the age of 12 (the youngest age at which we were able to link CLA records to ALSPAC).

Availability of cohort data

Maternal questionnaire response rates were highest for participants with no social care record and lowest for those with a CLA record at all time points. Differences generally widened over time (Figure 2). Patterns were similar for partner and child, but not teacher, questionnaires (supplementary Figures 1a-d).

Educational outcomes at 16 years

Of the 9545 children in these analyses, 49 had CLA and 64 CIN (no CLA) records during KS4. These groups were more disadvantaged than their peers in early-life and during KS4 (Table 1). They were more likely to have joined their school recently.

Of those with CIN or CLA records, <15% passed 5+ good GCSEs compared to >50% of their peers. Mean percentage scores were also markedly lower (Table 2). They were more likely to have SEN and persistent absence rates were higher, particularly for the in need group. Adjustment for school absence, neighbourhood deprivation, and receipt of FSM attenuated associations slightly for the CIN group but had less of an impact for the CLA group (Table 3). Adjustment for early-life maternal and SEP factors had more of an attenuating effect for the CLA than the CIN group. Attainment differences between these groups and their peers remained in the fully adjusted model.

In sensitivity analyses, when social care records at any time were considered, patterns were similar for the CLA group (n=76), while the CIN group (n=148) tended to do better than when restricted to only those who were in need during KS4 (Table 2). When the sample

For peer review only - http://bmjopen.bmj.com/site/about/guidelines.xhtml

UOB Confidential & Sensitive

excluded those with SEN or disability, results were similar to those of the main analyses (supplementary tables F and G).

Estimates of the relationship between social care characteristics and attainment were imprecise due to small numbers. Those in foster placements had higher capped percentage scores (mean 37.0, 95% CI 30.7 to 43.2, n=64) than those in children's/residential homes/residential schools (28.3, 14.7 to 42.0, n=12). With regards need status, 'child disability' was associated with the lowest attainment for the CIN group and 'socially unacceptable behaviour' for the CLA group. For both CLA and CIN groups, those in the 'parental illness/disability' category had the highest attainment. However confidence intervals were wide and overlapping.

For peer review only - http://bmjopen.bmj.com/site/about/guidelines.xhtml

UOB Confidential & Sensitive

Table 1: Summary of maternal, family and child characteristics, by social care status of child during Key Stage 4

		Child's social care status during KS4		
		No CLA/CIN record n=9432	CIN (no CLA) record n=64	CLA record n=49
**Maternal and family characteristics during pregnancy**[1]		% (95% CI)		
Maternal age (at delivery)	<=23 years	18.4 (17.7-19.2)	39.1 (27.0-51.4)	28.6 (15.4-41.7)
	>33 years	12.3 (11.6-12.9)	7.8 (1.1-14.6)	14.3 (4.1-24.5)
Relationship status	Married	75.0 (74.1-75.9)	53.8 (40.8-66.7)	49.5 (34.3-64.8)
	Resident partner	16.5 (15.7-17.3)	17.0 (6.9-27.1)	27.4 (13.5-41.3)
	Non-resident/no partner	8.5 (7.9-9.1)	29.2 (17.3-41.2)	23.1 (9.9-36.3)
Highest maternal education	A Level or degree	30.7 (29.8-31.7)	10.8 (2.3-19.3)	11.1 (0.9-21.4)
	O Level	36.5 (35.5-37.5)	40.7 (27.2-54.1)	26.3 (12.1-40.6)
	Vocational/none	32.8 (31.8-33.8)	48.5 (34.6-62.5)	62.5 (47.2-77.9)
Financial difficulties	Highest quartile	21.2 (20.3-22.1)	42.2 (27.6-56.8)	46.8 (30.4-63.2)
Housing tenure	Owned/mortgaged	73.7 (72.8-74.7)	54.3 (41.4-67.3)	33.7 (19.2-48.2)
Maternal smoking	Yes	26.6 (25.7-27.6)	41.3 (28.2-54.3)	58.6 (42.3-75.0)
Maternal alcohol - first trimester, ≥1 unit per week	Yes	15.2 (14.4-15.9)	17.8 (7.6-28.0)	21.7 (8.0-35.3)
Maternal alcohol - 2nd trimester, ever ≥4 units in one day	Yes	16.9 (16.1-17.6)	26.7 (14.8-38.5)	21.1 (7.6-34.6)
Depression score	Highest quartile	23.4 (22.5-24.3)	29.4 (16.4-42.4)	47.8 (31.5-64.1)
Low social support	Yes	10.3 (9.6-11.0)	20.8 (8.7-32.8)	25.9 (10.6-41.2)
**Child, school and neighbourhood characteristics during KS4**[1]		% or mean (95% CI)		
Sex	Female	49.6 (48.6-50.6)	51.6 (39.0-64.2)	49.0 (34.5-63.5)
Age at start of Year 11	Mean (years)	15.5 (15.4-15.5)	15.5 (15.4-15.6)	15.5 (15.4-15.5)
In receipt of free school meals	Yes	6.1 (5.6-6.6)	26.6 (15.4-37.7)	10.2 (1.4-19.0)
Joined school during KS4	Yes	1.4 (1.1-1.6)	7.8 (1.1-14.6)	12.2 (2.7-21.8)
Neighbourhood deprivation (IDACI)	Low, <10%	43.9 (42.9-44.9)	20.3 (10.2-30.4)	28.6 (15.4-41.7)
	High, >=40%	10.1 (9.5-10.7)	25.0 (14.1-35.9)	20.4 (8.7-32.1)

[1] For brevity, not all categories are presented for each categorical variable.

For peer review only - http://bmjopen.bmj.com/site/about/guidelines.xhtml
UOB Confidential & Sensitive
For peer review only

Table 2: Educational attainment, persistent absence, and special educational needs by child social care status

	Social care status during KS4			Social care status any time		
	No CLA/CIN record n=9432	CIN (no CLA) record n=64	CLA record n=49	No CLA/CIN record n=9321	CIN (no CLA) record n=148	CLA record n=76
**Educational Attainment**	% or mean (95% CI)			% or mean (95% CI)		
5+ A*-C GCSEs including English & Maths	53.0 (52.0-54.0)	10.9 (3.1-18.8)	12.2 (2.7-21.8)	53.3 (52.3-54.4)	19.6 (13.1-26.1)	10.5 (3.5-17.6)
Capped percentage point score	68.9 (68.5-69.3)	37.4 (31.3-43.5)	34.9 (27.4-42.3)	69.1 (68.7-69.5)	47.7 (43.8-51.7)	33.9 (28.2-39.6)
**Special Educational Needs (SEN)** [1]						
School action	8.5 (7.9-9.1)	12.5 (4.2-20.8)	n<5	8.4 (7.8-9.0)	16.2 (10.2-22.2)	9.2 (2.6-15.9)
School action plus	3.1 (2.8-3.5)	15.6 (6.5-24.8)	24.5 (12.0-37.0)	3.1 (2.7-3.4)	8.1 (3.7-12.6)	21.1 (11.7-30.4)
Statement of Special Educational Needs	2.4 (2.1-2.7)	46.9 (34.3-59.4)	24.5 (12.0-37.0)	2.3 (2.0-2.6)	22.3 (15.5-29.1)	35.5 (24.5-46.5)
**Persistent absence**	6.8 (6.3-7.3)	32.8 (21.0-44.6)	18.4 (7.1-29.6)	6.7 (6.2-7.2)	19.6 (13.1-26.1)	21.1 (11.7-30.4)

[1]For definitions of these SEN categories, please see Supplementary Text.

For peer review only

For peer review only - http://bmjopen.bmj.com/site/about/guidelines.xhtml

UOB Confidential & Sensitive

Table 3: Association between child social care status and educational outcomes, with adjustment for early-life and KS4 variables

Attainment Outcome	Care status during KS4	Model 1[1] (Age and Sex)	Model 2[2] (KS4 variables)	Model 3[3] (Early-life variables)	Model 4[4] (Fully adjusted)
		OR (95% CI)	OR (95% CI)	OR (95% CI)	OR (95% CI)
5+ A*-C GCSEs including English & Maths	Not CIN or CLA	Ref	Ref	Ref	Ref
	CIN (not CLA)	0.11 (0.05-0.27)	0.17 (0.07-0.40)	0.15 (0.06 to 0.36)	0.19 (0.08-0.46)
	CLA	0.14 (0.05-0.35)	0.14 (0.06-0.36)	0.25 (0.10 to 0.63)	0.24 (0.09-0.63)
		Coeff (95% CI)	Coeff (95% CI)	Coeff (95% CI)	Coeff (95% CI)
Capped percentage point score	Not CIN or CLA	Ref	Ref	Ref	Ref
	CIN (not CLA)	-22.1 (-26.7 to -17.5)	-14.1 (-18.4 to -9.8)	-18.4 (-22.6 to -14.1)	-13.1 (-17.1 to -9.0)
	CLA	-28.4 (-33.5 to -23.3)	-25.0 (-29.7 to -20.3)	-21.9 (-26.6 to -17.2)	-20.6 (-25.0 to -16.1)

[1]Adjusted for child age and sex
[2]Adjusted for child age and sex, plus KS4 time-point variables (persistent school absence, in receipt of free school meals, school mobility, IDACI of residential neighbourhood)
[3]Adjusted for child age and sex, plus early-life [maternal and SEP] variables (maternal age at delivery, education, partner status, housing tenure, financial difficulties, smoking, alcohol, depression, social support)
[4]Adjusted for child age and sex, plus KS4 and early life variables

For peer review only

For peer review only - http://bmjopen.bmj.com/site/about/guidelines.xhtml

UOB Confidential & Sensitive

DISCUSSION

Children who were looked-after or in need during KS4 had low attainment at age 16. The early-life exposures we considered were not a major explanatory factor. We believe this is the first time linkage to the CLA Return and CIN Census has been used to identify birth cohort participants who were looked-after or in need during adolescence. As linkage data were also used for outcome measures, participants could be included even if their families no longer actively participated in the cohort study. Record linkage therefore allowed vulnerable children to not only be included in research but to be the focus of it. However, the identification and inclusion of in need and looked-after children in research using record linkage does have challenges.

For cohort studies in England with relevant permissions, linkage to the CLA Return and CIN Census via the NPD offers a convenient means of identifying participants who have been in need or looked-after. For cohorts younger than ALSPAC, this method would allow identification of social care records that cover most, if not all, of participants' childhoods. However, in ALSPAC we were only able to link to records covering a period during adolescence. Consequently, outcomes at younger ages cannot be examined by social care status in ALSPAC using this method. Of the looked-after children in England the same age as the ALSPAC participants, we found around 40% had left the care system by the age of 12. Consequently, our reference group likely includes children who were looked-after or in need at younger ages only.

Examination of questionnaire response rates showed the value of using linked outcome data to increase the inclusion of vulnerable children in research: there was little questionnaire data available beyond infancy for participants with social care records in adolescence. In this current study, we examined educational outcomes at age 16, obtained from the NPD. The

For peer review only - http://bmjopen.bmj.com/site/about/guidelines.xhtml

UOB Confidential & Sensitive

association between social care status and other later outcomes available from linked data could also be investigated using ALSPAC, such as mental illness or entry into higher education.

ALSPAC participants with CLA and CIN records in adolescence had lower educational attainment than their peers in the reference group. In the most recent national data available, attainment patterns by social care status broadly reflect these findings(1). We found persistent absence rates to be considerably lower for those looked-after than those in need during KS4. Similarly, in the national data (on pupils of all ages) 9% of looked-after children were persistent absentees and 28% of children in need(1). Therefore, although our participants were in KS4 around 10 years ago and the number with social care records small, the patterning of educational characteristics by care status is broadly similar to the present-day situation.

Using both ALSPAC questionnaire data and measures from the NPD, we found a persistence of disadvantage from early-life to adolescence for participants with CIN and CLA records. Social disadvantage is known to be strongly associated with poorer educational attainment(30, 31), and our SEP measures were strongly related to the educational outcomes. Adjustment for them attenuated associations slightly but the low attainment of the CLA and CIN groups remained. We are not considering the SEP measures as confounders but rather part of the complex causal pathway from early-life adversity through to poor educational attainment. It is notable that many of the mothers of the children with social care records had very low educational attainment themselves.

Alcohol and tobacco, the most commonly used substances in pregnancy, can cross the placenta and alter normal brain development (32). In our sample, those with social care records had higher levels of exposure to these substances than their peers. Those with CIN

For peer review only - http://bmjopen.bmj.com/site/about/guidelines.xhtml

UOB Confidential & Sensitive

records were the most likely to have ever been exposed to ≥4 units of alcohol in one day. Exposure to this level of alcohol has previously been found to be negatively associated with educational attainment in the ALSPAC sample (33, 34). However, in our analyses, adjustment for maternal alcohol use did not alter the associations observed between social care status and educational attainment. This may be due in part to our binary alcohol measures (necessary due to small numbers) failing to accurately capture exposure, and not identifying those at highest risk. This is an important limitation as many children in the care system have foetal alcohol syndrome, a condition which is often undiagnosed and is the most common, non-genetic cause of learning disability in the UK (35, 36). The majority of participants with CLA records had a mother who smoked during pregnancy, and this exposure was negatively associated with attainment. However there is debate as to whether maternal smoking during pregnancy is a direct cause of poorer child educational attainment, or is instead a strong marker of socio-economic disadvantage (37-39).

Overall, little of the poor educational outcomes in the looked-after and in need groups appeared to be explained by the early-life exposures we considered. This could suggest there is scope for later experiences, including social care, to improve outcomes. However, other early-life exposures, or genetic factors, that we have not considered could be of importance.

While aspects of care itself could be important contributors to educational outcomes, ascertaining direction of causality in the relationship between child behaviours, care characteristics, and educational outcomes is difficult. As expected, we found children in foster care had higher attainment than those not in family-based care: the latter children are likely to be those whose foster placements have broken down, reflecting complex additional needs and challenging behaviours. Further, foster carers may have greater commitment and longer-term interest in the child than group care staff(40). We were unable to consider placement stability, which has previously been shown to be beneficial(41). However, in

For peer review only - http://bmjopen.bmj.com/site/about/guidelines.xhtml

UOB Confidential & Sensitive

For peer review only

concordance with previous studies, school mobility was associated with lower attainment(42, 43) and children with CLA or CIN records were much more likely to have changed school during KS4 than their peers.

The relatively high proportion of looked-after and in need children with SEN or disability did not appear to explain the low average attainment of these groups. Similarly, in the national data looked-after children with no identified SEN made less educational progress than non-looked-after children(1). It is important to note that the attainment gap between looked-after and in need children and their peers is apparent from a young age, often before the child enters care(1, 41). Being looked-after may not be the principal cause of poor attainment, rather it is a marker of extreme childhood adversity, which is itself associated with poor outcomes. Being in care is often beneficial for a child's education(17, 18, 41).

Strengths of this study include the use of a novel method to identify vulnerable adolescents in a population-based cohort, and objective outcome measures. Limitations include incomplete ascertainment of social care record status, little cohort data beyond early childhood for those with social care records, and small numbers. Children who experience the most disadvantaged starts in life are likely under-represented in ALSPAC as their mothers would have been least likely to attend antenatal appointments, which is where many mothers were recruited to the study.

Conclusions

Data linkage provides a means of identifying children with social services contact in cohort studies and of increasing their inclusion in research. The poor educational outcomes of the ALSPAC adolescents with social care records did not appear to be substantially explained by the early-life exposures we considered. Further research, ideally with social care data across the lifecourse, would help identify which factors are important in explaining the poor

For peer review only - http://bmjopen.bmj.com/site/about/guidelines.xhtml

UOB Confidential & Sensitive

educational attainment of these vulnerable children, and would help inform the development of effective interventions.

For peer review only - http://bmjopen.bmj.com/site/about/guidelines.xhtml

UOB Confidential & Sensitive

Acknowledgments

We are extremely grateful to all the families who took part in this study, the midwives for their help in recruiting them, and the whole ALSPAC team, which includes interviewers, computer and laboratory technicians, clerical workers, research scientists, volunteers, managers, receptionists and nurses.

Conflicts of interest

JM is a foster carer. AT, AB and DW have no conflicts of interest to declare.

Funding

Core support for ALSPAC is provided by the UK Medical Research Council and Wellcome (Grant reference: 102215/2/13/2) and the University of Bristol. The Wellcome Trust (WT086118) funded ALSPACs linkage infrastructure through the Project to Enhance ALSPAC through Record Linkage (PEARL). This work was supported by the Elizabeth Blackwell Institute for Health Research, University of Bristol and the Wellcome Trust Institutional Strategic Support Fund (Grant number: 105612/Z/14/Z).

Contributor Statement

JM and AB conceived the study, and AT and JM developed the research question. AT conducted the analyses, interpreted the data and drafted the manuscript. JM and DW helped interpret the data and critically revised the paper. AB critically revised the paper.

Data Sharing Statement

The ALSPAC data management plan (available here: http://www.bristol.ac.uk/alspac/researchers/data-access/ documents/alspac-datamanagement-plan.pdf) describes in detail the policy regarding data sharing, which is through a system of managed open access.

For peer review only

UOB Confidential & Sensitive

REFERENCES

1. Department for Education, National Statistics. Outcomes for Children Looked After by Local Authorities in England, as at 31 March 2016 (SRF 12/2017). 2017 [Available from: https://www.gov.uk/government/uploads/system/uploads/attachment_data/file/602087/SFR12_2017_Text.pdf.

2. Ford T, Vostanis P, Meltzer H, Goodman R. Psychiatric disorder among British children looked after by local authorities: comparison with children living in private households. The British Journal of Psychiatry. 2007;190(4):319-25.

3. Oswald SH, Heil K, Goldbeck L. History of Maltreatment and Mental Health Problems in Foster Children: A Review of the Literature. Journal of Pediatric Psychology. 2010;35(5):462-72.

4. Ward J, Henderson Z, Pearson G. One problem among many: drug use among care leavers in transition to independent living 2003 [Available from: http://www.drugsandalcohol.ie/5584/1/Home_Office_Research_Study_260_One_problem_among_many.pdf.

5. Viner RM, Taylor B. Adult Health and Social Outcomes of Children Who Have Been in Public Care: Population-Based Study. Pediatrics. 2005;115(4):894-9.

6. Botchway SK, Quigley MA, Gray R. Pregnancy-associated outcomes in women who spent some of their childhood looked after by local authorities: findings from the UK Millennium Cohort Study. BMJ Open. 2014;4(12).

7. Bellis MA, Lowey H, Leckenby N, Hughes K, Harrison D. Adverse childhood experiences: retrospective study to determine their impact on adult health behaviours and health outcomes in a UK population. Journal of Public Health. 2014;36(1):81-91.

8. Hughes K, Lowey H, Quigg Z, Bellis MA. Relationships between adverse childhood experiences and adult mental well-being: results from an English national household survey. BMC Public Health. 2016;16(1):222.

9. Plant DT, Jones FW, Pariante CM, Pawlby S. Association between maternal childhood trauma and offspring childhood psychopathology: mediation analysis from the ALSPAC cohort. The British Journal of Psychiatry. 2017.

10. Teyhan A, Wijedasa D, Macleod J. Adult psychosocial outcomes of men and women who were looked-after or adopted as children: prospective observational study. BMJ Open. 2018.

11. Children's commissioner for England. On measuring the number of vulnerable children in England 2017 [Available from: https://www.childrenscommissioner.gov.uk/wp-content/uploads/2017/07/CCO-On-vulnerability-Overveiw-2.pdf.

12. Mezey G, Robinson F, Campbell R, Gillard S, Macdonald G, Meyer D, et al. Challenges to undertaking randomised trials with looked after children in social care settings. Trials. 2015;16(1):206.

13. Department for Education. Children in need census 2017 to 2018. Guide for local authorities - version 1.2 2018 [Available from: https://assets.publishing.service.gov.uk/government/uploads/system/uploads/attachment_data/file/695113/CIN17-18_Guide_v1.2-web_version.pdf.

14. Department for Education. Characteristics of children in need: 2016 to 2017 England (SFR 61/2017)2017 Accessed February 2018. Available from: https://www.gov.uk/government/uploads/system/uploads/attachment_data/file/656395/SFR61-2017_Main_text.pdf.

15. Department for Education, National Statistics. Children looked after in England (including adoption), year ending 31 March 2017 (SFR 20/2017)2017 Accessed February 2018. Available from: https://www.gov.uk/government/uploads/system/uploads/attachment_data/file/664995/SFR50_2017-Children_looked_after_in_England.pdf.

16. Fletcher J, Strand S, Thomas S. The Educational Progress of Looked After Children in England.

For peer review only - http://bmjopen.bmj.com/site/about/guidelines.xhtml

UOB Confidential & Sensitive

Technical Report 1: Secondary School Progress and Attainment 2015 [Available from: http://reescentre.education.ox.ac.uk/wordpress/wp-content/uploads/2015/11/EducationalProgressLookedAfterChildrenTechnical-Report-1_Nov2015.pdf.
17. Sebba J, Berridge D, Luke N, Fletcher J, Bell K, Strand S, et al. The Educational Progress of Looked After Children in England: Linking Care and Educational Data 2015 [Available from: http://reescentre.education.ox.ac.uk/wordpress/wp-content/uploads/2015/11/EducationalProgressLookedAfterChildrenOverviewReport_Nov2015.pdf.
18. Berridge D. Educating young people in care: What have we learned? Children and Youth Services Review. 2012;34(6):1171-5.
19. Boyd A, Golding J, Macleod J, Lawlor DA, Fraser A, Henderson J, et al. Cohort Profile: The 'Children of the 90s'—the index offspring of the Avon Longitudinal Study of Parents and Children. International Journal of Epidemiology. 2012.
20. Fraser A, Macdonald-Wallis C, Tilling K, Boyd A, Golding J, Davey Smith G, et al. Cohort Profile: The Avon Longitudinal Study of Parents and Children: ALSPAC mothers cohort. International Journal of Epidemiology. 2013;42(1):97-110.
21. ALSPAC. Data Dictionary [Available from: http://bristol.ac.uk/alspac/researchers/data-access/data-dictionary/.
22. Boyd A, Tilling K, Cornish R, Davies A, Humphries K, Macleod J. Professionally designed information materials and telephone reminders improved consent response rates: evidence from an RCT nested within a cohort study. Journal of Clinical Epidemiology. 2015;68(8):877-87.
23. Mc Grath-Lone L, Harron K, Dearden L, Nasim B, Gilbert R. Data Resource Profile: Children Looked After Return (CLA). International Journal of Epidemiology. 2016;45(3):716-7f.
24. Emmott E, Jay M, Woodman J. Cohort Profile: Children in Need Census (CIN) Records of Vulnerable Children in England (pre-print). BMJ Open. 2018.
25. Department for Education. The National Pupil Database: User Guide 2015 [Available from: www.gov.uk/government/uploads/system/uploads/attachment_data/file/472700/NPD_user_guide.pdf.
26. National Pupil Database in England Wiki. KS4 2016 [Available from: https://nationalpupildatabase.wikispaces.com/KS4.
27. Thorpe K. A study of the use of the Edinburgh postnatal depression scale with parent groups outside the postpartum period. Journal of Reproductive and Infant Psychology. 1993;11(2):119-25.
28. GOV.UK. Apply for free school meals 2018 [Available from: https://www.gov.uk/apply-free-school-meals.
29. Department for Communities and Local Government. The English Indices of Deprivation 2015 [Available from: https://www.gov.uk/government/uploads/system/uploads/attachment_data/file/465791/English_Indices_of_Deprivation_2015_-_Statistical_Release.pdf.
30. Hirsch D. Experiences of poverty and educational disadvantage 2007 [Available from: https://www.jrf.org.uk/report/experiences-poverty-and-educational-disadvantage.
31. Goodman A, Gregg P. Poorer children's educational attainment: how important are attitudes and behaviour? 2010 [Available from: https://www.jrf.org.uk/report/poorer-children%E2%80%99s-educational-attainment-how-important-are-attitudes-and-behaviour.
32. Scott-Goodwin AC, Puerto M, Moreno I. Toxic effects of prenatal exposure to alcohol, tobacco and other drugs. Reproductive Toxicology. 2016;61:120-30.
33. Sayal K, Heron J, Draper E, Alati R, Lewis SJ, Fraser R, et al. Prenatal exposure to binge pattern of alcohol consumption: mental health and learning outcomes at age 11. European child & adolescent psychiatry. 2014;23(10):891-9.
34. Alati R, Davey Smith G, Lewis SJ, Sayal K, Draper ES, Golding J, et al. Effect of Prenatal Alcohol Exposure on Childhood Academic Outcomes: Contrasting Maternal and Paternal Associations in the ALSPAC Study. PLOS ONE. 2013;8(10):e74844.

For peer review only - http://bmjopen.bmj.com/site/about/guidelines.xhtml

UOB Confidential & Sensitive

35. McQuire C, Mukherjee R, Hurt L, Higgins A, Greene G, Farewell D, et al. Screening prevalence of fetal alcohol spectrum disorders in a region of the United Kingdom: A population-based birth-cohort study. Preventive Medicine. 2019;118:344-51.
36. Bakhireva LN, Garrison L, Shrestha S, Sharkis J, Miranda R, Rogers K. Challenges of diagnosing fetal alcohol spectrum disorders in foster and adopted children. Alcohol. 2018;67:37-43.
37. Kristjansson AL, Thomas S, Lilly CL, Thorisdottir IE, Allegrante JP, Sigfusdottir ID. Maternal smoking during pregnancy and academic achievement of offspring over time: A registry data-based cohort study. Preventive Medicine. 2018;113:74-9.
38. Lambe M, Hultman C, Torr, xe, ng A, MacCabe J, et al. Maternal Smoking during Pregnancy and School Performance at Age 15. Epidemiology. 2006;17(5):524-30.
39. Knopik VS. Maternal smoking during pregnancy and child outcomes: real or spurious effect? Dev Neuropsychol. 2009;34(1):1-36.
40. Lo A, Roben CKP, Maier C, Fabian K, Shauffer C, Dozier M. “I want to be there when he graduates:” Foster parents show higher levels of commitment than group care providers. Children and Youth Services Review. 2015;51:95-100.
41. Luke N, Sinclair I, O'Higgins A. The Educational Progress of Looked After Children in England. Technical report 2: Relating Care to Educational Attainment and Progress 2015 [Available from: https://weblearn.ox.ac.uk/access/content/group/5cacf183-ce54-47f0-9f17-d4b0fe1cf292/EducationalProgressLookedAfterChildrenTechnical%20Report%202_Nov2015.pdf.
42. Hutchings HA, Evans A, Barnes P, Demmler J, Heaven M, Hyatt MA, et al. Do children who move home and school frequently have poorer educational outcomes in their early years at school? An anonymised cohort study. PloS one. 2013;8(8):e70601.
43. Strand S, Demie F. Pupil mobility, attainment and progress in secondary school. Educational Studies. 2007;33(3):313-31.

For peer review only

For peer review only - http://bmjopen.bmj.com/site/about/guidelines.xhtml

UOB Confidential & Sensitive

Figure Legends

Figure 1 – Flow Chart of Sample

Figure 2 - Maternal questionnaire response rates by child social care status

For peer review only

For peer review only - http://bmjopen.bmj.com/site/about/guidelines.xhtml

UOB Confidential & Sensitive

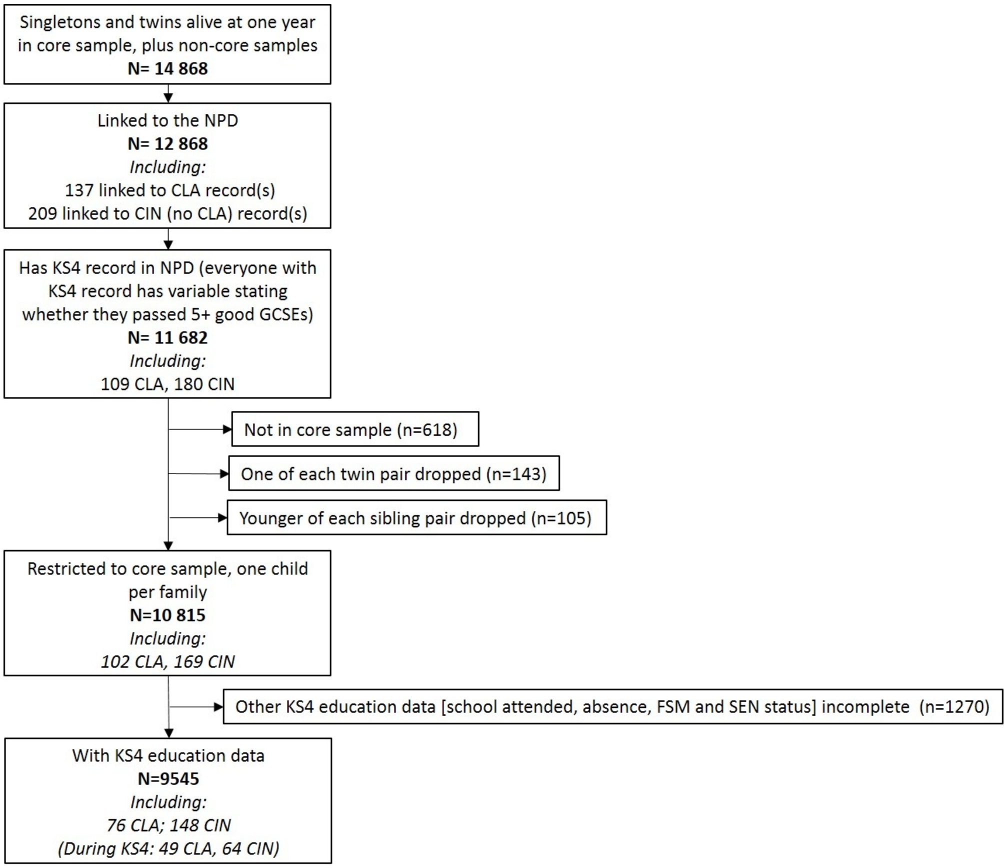

Figure 1 - Flow Chart of Sample

218x189mm (150 x 150 DPI)

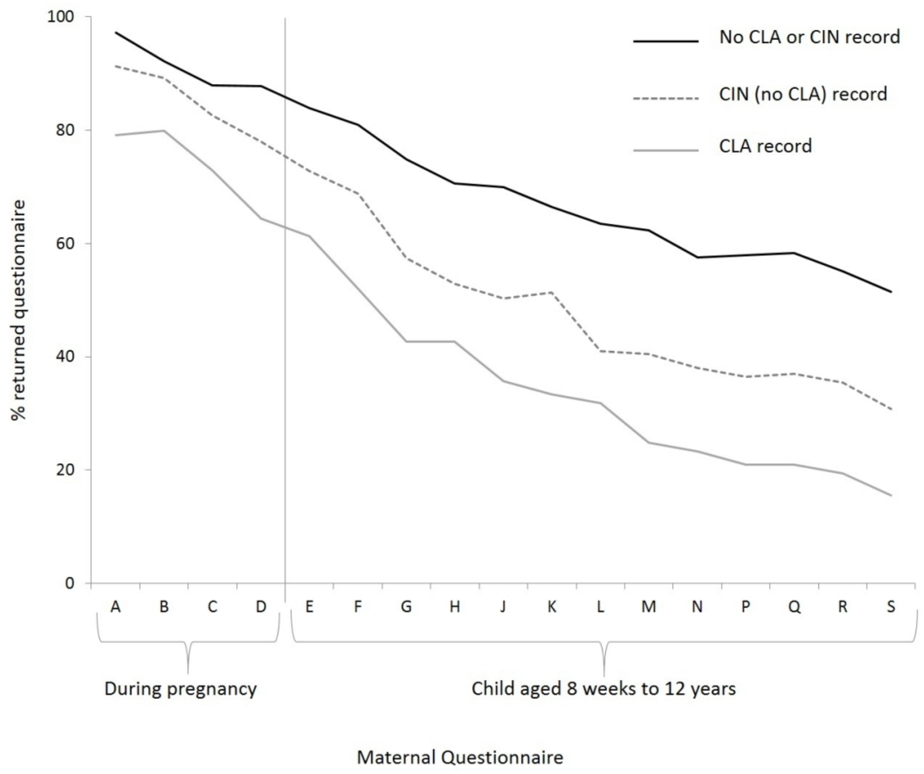

Notes on figure:

Sample restricted to mothers in the core sample, whose child has been linked to the National Pupil Database.

Mothers may not have completed every question within each questionnaire.

Figure 2 - Maternal questionnaire response rates by child social care status

231x223mm (150 x 150 DPI)

Supplementary (online only) Tables

Table A: Summary of CLA and CIN data availability by year and period of birth

Table B: Summary of early-life (maternal and family) variables before and after multiple imputation

Table C: Summary of CIN data for ALSPAC participants linked to a post-2008 CLA record

Table D: Summary of care data for ALSPAC participants linked to a post-2005 CLA record

Table E: Comparison of care characteristics between ALSPAC CLA participants and CLA individuals in general population (in ALSPAC area and in England) of same age.

Table F: Educational attainment, absence, and special educational needs by care status – no SEN

Table G: Educational attainment, absence, and special educational needs by care status – no disability

For peer review only

Table A: Summary of CLA and CIN data availability by year and period of birth

	Expected school year in March of each year[1]					
Period of birth	2006[2]	2007[2]	2008[2]	2009[2,3]	2010[2,3]	2011[2,3]
April 1991 to August 1991	Year 10	**Year 11[4]**	Year 12	Year 13		
September 1991 to August 1992	Year 9	Year 10	**Year 11[4]**	Year 12	Year 13	
September 1992 to January 1993	Year 8	Year 9	Year 10	**Year 11[4]**	Year 12	Year 13

[1] The school year in England runs from September to July. In contrast, the CLA and CIN data collection year runs from April to March. For example, the 2006 CLA dataset would cover the period from April 2005 to March 2006; the oldest ALSPAC participants would have been in Year 10 in March 2006, and the youngest in Year 8.

[2] CLA data linked to NPD available

[3] CIN data linked to NPD available

[4] GCSE exams are taken in May/June of Year 11.

Determining who had CIN record during KS4:

For the CIN data, the youngest cohort participants were in Year 11 at the time of the earliest CIN data collection and they were coded as being 'in need during KS4' if they had a CIN record. However, the majority of the cohort were already in Year 12 or 13 at this time, and so we calculated the age they had been referred, and identified those who had been referred before they sat their KS4 exams (age on the 1st June of the year they were in Year 11 was used as a proxy for age that exams were taken).

For peer review only

Table B: Summary of early-life (maternal and family) variables before and after multiple imputation

Variables with missing data in study sample[1]	% missing	Categories[2]	Study sample [N=9545]	
			Before imputation [N specified for each variable individually]	Imputed [N=9545]
**Variables reported by mother during pregnancy**				
Relationship status	5.2		N=9048	
		Married (%)	75.7 (74.8-76.6)	74.7 (73.8-75.6)
		Resident partner (%)	16.1 (15.3-16.8)	16.5 (15.8-17.3)
		Non-resident/no partner (%)	8.2 (7.7-8.8)	8.7 (8.1-9.3)
Highest maternal education	9.7		N=8623	
		A Level or degree (%)	31.7 (30.8-32.7)	30.5 (29.5-31.4)
		O Level	36.8 (35.8-37.9)	36.5 (35.5-37.5)
		Vocational/none (%)	31.4 (30.4-32.4)	33.0 (32.0-34.0)
Financial difficulties	12.1		N=8387	
		Q1 (none) (%)	34.9 (33.9-35.9)	33.7 (32.7-34.7)
		Q4 (high) (%)	20.4 (19.5-21.3)	21.5 (20.6-22.4)
Housing tenure	5.7		N=9003	
		Owned/mortgaged (%)	74.6 (73.6-75.4)	73.4 (72.5-74.3)
Maternal smoking	5.0		N=9068	
		Yes (%)	26.2 (25.3-27.1)	26.9 (26.0-27.8)
Depression score	13.1		N=8294	
		Highest quartile (%)	22.7 (21.8-23.6)	23.5 (22.6-24.5)
Frequency of alcohol drinking in first trimester	6.0		N=8975	
		Never or <1 unit/week	84.8 (84.1-85.6)	84.8 (84.1-85.5)
		1-6 units or 7+ units/week	15.2 (14.4-15.9)	15.2 (14.5-15.9)
Any days drinking ≥4 units alcohol during second trimester	6.2		N=8950	
		Yes	16.8 (16.0-17.5)	16.9 (16.2-17.7)
Low social support	16.8		N=7942	
		Yes (%)	9.3 (8.7-10.0)	10.5 (9.8-11.2)
**Educational attainment from NPD**				
Capped percentage point score[3]	0.9	Mean	68.8 (68.4-69.2)	68.5 (68.1-68.9)

[1] The following variables had no missing data in study sample: child age, sex, maternal age at delivery, attainment of 5+ good GCSEs, persistent absence, SEN status, school attended.

[2] For brevity, not all categories are presented for each variable.

[3] The binary attainment variable (5+ good GCSEs) was complete for all those with a KS4 NPD record, but a small number (n=82) had missing capped point score data.

For peer review only

Table C: Summary of CIN data for ALSPAC participants linked to a post-2008 CIN record (but no CLA record)

Variable		CIN (no CLA) record N=209
Age (yrs) at referral date	Median (range)	16.7 (2.5-18.1)
Primary need status	Abuse or neglect (%, 95% CI)	22.0 (16.9-28.2)
	Child disability/illness (%, 95% CI)	23.4 (18.2-29.7)
	Parental illness/disability (%, 95% CI)	[n<5]
	Family in acute stress (%, 95% CI)	21.5 (16.4-27.7)
	Family dysfunction (%, 95% CI)	18.2 (13.5-24.1)
	Socially unacceptable behaviour (%, 95% CI)	[n<5]
	Low income (%, 95% CI)	[n<5]
	Absent parenting (%, 95% CI)	[n<5]
	Cases other than CIN (%, 95% CI)	[n<5]
	Not stated (%, 95% CI)	10.0 (6.6-15.0)
Child ever coded as having disability	Yes (%, 95% CI)	22.0 (16.9-28.2)

For peer review only

Table D: Summary of care data for ALSPAC participants linked to a post-2005 CLA record

		Linked to CLA record		
Variable		Overall<br><br>n=137	Eligible for one-third sample[1]<br>n=47	Ineligible for one-third sample[2]<br>n=90
Total number of periods of care[3]	Median, range	1 (1-13)	1 (1-8)	1 (1-13)
Total number of episodes of care[3]	Median, range	3 (1-28)	3 (1-24)	3 (1-28)
Age (yrs) at start of first period of care[3]	Median, range	13.2 (0-17.8)	11.5 (0-17.6)	13.7 (0.1-17.8)
Age (yrs) at end of last episode of care[3]	Median, range	17.7 (13.1-18.0)	18.0 (13.1-18.0)	17.1 (13.2-18.0)
		N=134	N=45	N=89
Total duration (days) in care [3,4]	Median, range	906 (1-5736)	1394 (2-5498)	604 (1-5736)
Primary need category at start of first period of care[3,5]	Abuse or neglect (%, 95% CI)	30.7 (23.4-39.0)	34.4 (21.6-49.1)	28.9 (20.3-39.3)
	Child disability (%, 95% CI)	16.1 (10.8-23.3)	19.1 (10.0-33.5)	14.4 (8.5-23.5)
	Parental illness/disability (%, 95% CI)[6]	4.4 (2.0-9.5)	/	/
	Family in acute stress (%, 95% CI)	18.2 (12.6-25.7)	17.0 (8.5-31.1)	18.9 (12.0-28.5)
	Family dysfunction (%, 95% CI)	17.5 (12.0-24.9)	25.5 (14.8-40.4)	13.3 (7.6-22.2)
	Socially unacceptable behaviour (%, 95% CI)[6]	8.8 (5.0-14.9)	/	/
	Absent parenting (%, 95% CI)[6]	4.4 (2.0-9.5)	/	/
Placement type for last episode of care	Foster care (%, 95% CI)	62.8 (54.3-70.5)	57.4 (42.6-71.1)	65.6 (55.0-74.8)
	Children's/residential/care home or school (%, 95% CI)	18.2 (12.6-25.7)	23.4 (13.2-38.1)	15.6 (9.3-24.8)
	Other (%, 95% CI)	19.0 (13.2-26.5)	19.1 (10.0-33.5)	18.9 (12.0-28.5)
Reason for last episode of care ending	Returned home to parents or relatives (%, 95% CI)	36.5 (28.8-45.0)	27.7 (16.5-42.6)	41.1 (31.3-51.7)
	Moved to independent living (%, 95% CI)	16.8 (11.4-24.1)	19.1 (10.0-33.4)	15.6 (9.3-24.8)
	Residential care funded by adult social services (%, 95% CI)	13.1 (8.3-20.0)	21.3 (11.6-35.8)	8.9 (4.4-17.0)
	Other (%, 95% CI)	32.8 (25.4-41.2)	31.9 (19.9-47.0)	34.4 (25.2-45.0)

[1]Individuals eligible for the one-third sample will have CLA records in the period 1998-2003 if they were looked-after during this time.
[2] No CLA data were collected from 2008-2003 for those ineligible for the one-third sample; therefore their looked-after status during this period is unknown.
[3] Measures relate to the data available from linkage only: these should be complete for those eligible for the one-third sample, but will not be for those in the ineligible sample who were looked-after during 1998-2003.
[4]The sum of the duration of all episodes of care, which may or may not have been consecutive.
[5]Refers to primary need at the start of the first period of care for which we have a record.
[6]Percentages not shown by one-third sample status for these need categories to prevent derivation of small cell counts (n<5).

Table E: Comparison of care characteristics between ALSPAC participants with CLA records and individuals with CLA records in general population of same age (in ALSPAC area and in England).

		Born 1991 or 1992 with CLA record in England[1]<br><br>n=43938	Born 1991 or 1992 with CLA record in ALSPAC area[1,2]<br><br>n=713	ALSPAC participants with CLA record<br><br>n=137
Total number of periods of care [3]	Median, range	1 (1-516)	1 (1-46)	1 (1-13)
Total number of episodes of care[3]	Median, range	2 (1-517)	2 (1-49)	3 (1-28)
Age (yrs) at start of first period of care[3]	Median, range	9.7 (0-18)	7.6 (0-18)	13.2 (0-17.8)
Age (yrs) at end of last episode of care[3]	Median, range	15.5 (0-19.9)	14.2 (0-18.2)	17.7 (13.1-18.0)
Last period of care ended <12yrs	Yes (%, 95% CI)	35.6 (35.2-36.1)	41.9 (38.4-45.6)	0
		n=43554	n=707	n=134
Total duration (days) in care [3,4]	Median, range	461 (1-6575)	427 (1-6069)	906 (1-5736)
		n=30250	n=453	n=137
Primary need category at start of first period of care[3,5]	Abuse or neglect (%, 95% CI)	35.3 (34.8-35.9)	26.3 (22.4-30.5)	30.7 (23.4-39.0)
	Child disability (%, 95% CI)	12.7 (12.4-13.1)	16.1 (13.0-19.8)	16.1 (10.8-23.3)
	Parental illness/disability (%, 95% CI)	3.9 (3.7-4.1)	5.1 (3.4-7.5)	4.4 (2.0-9.5)
	Family in acute stress (%, 95% CI)	10.9 (10.5-11.2)	18.5 (15.2-22.4)	18.2 (12.6-25.7)
	Family dysfunction (%, 95% CI)	12.4 (12.0-12.8)	20.5 (17.0-24.5)	17.5 (12.0-24.9)
	Socially unacceptable behaviour (%, 95% CI)	6.3 (6.1-6.6)	6.2 (4.3-8.8)	8.8 (5.0-14.9)
	Absent parenting (%, 95% CI)	18.1 (17.7-18.6)	7.3 (5.2-10.1)	4.4 (2.0-9.5)
		N=43602	N=712	N=137
Placement type for last episode of care	Foster care	32.8 (32.4-33.3)	42.0 (38.4-45.7)	62.8 (54.3-70.5)
	Children's/residential/care home/school	14.1 (13.7-14.4)	9.4 (7.5-11.8)	18.2 (12.6-25.7)
	Other	53.1 (52.6-53.6)	49.0 (44.9-52.3)	19.0 (13.2-26.5)
		N=39,647	N=644	N=137
Reason for last episode of care ending	Returned home to parents or relatives	21.9 (21.5-22.3)	24.8 (21.7-28.3)	36.5 (28.8-45.0)
	Moved to independent living	17.7 (17.4-18.1)	14.8 (12.2-17.7)	16.8 (11.4-24.1)
	Residential care funded by adult social services	4.8 (4.5-5.0)	7.6 (5.8-9.9)	13.1 (8.3-20.0)

[1]Excludes the 137 individuals identified as being in ALSPAC

[2]Includes only those in the care of one of the following local authorities: City of Bristol; Bath and North East Somerset; South Gloucestershire; North Somerset.

For peer review only

Table F: Educational attainment, absence, and special educational needs by care status excluding those with SEN (n=8145)

**Outcome**	**Care status during KS4**	**Model 1[1] (Age and Sex)**	**Model 2[2] (KS4 variables)**	**Model 3[3] (Early-life variables)**	**Model 4[4] (Fully adjusted)**
		OR (95% CI)	OR (95% CI)	OR (95% CI)	OR (95% CI)
5 A*-C GCSEs inc. Eng. & Maths	Not CIN or CLA	Ref	Ref	Ref	Ref
	CIN (not CLA)	0.04 (0.01-0.36)	0.08 (0.01-0.65)	0.08 (0.01-0.68)	0.11 (0.01-1.02)
	CLA	0.26 (0.09-0.77)	0.31 (0.10-0.98)	0.35 (0.11-1.10)	0.40 (0.12-1.31)
		Coeff (95% CI)	Coeff (95% CI)	Coeff (95% CI)	Coeff (95% CI)
Capped percentage score	Not CIN or CLA	Ref	Ref	Ref	Ref
	CIN (not CLA)	-24.1 (-31.8 to -16.5)	-10.6 (-17.6 to -3.6)	-18.0 (-25.0 to -10.9)	-8.6 (-15.2 to -2.0)
	CLA	-26.7 (-33.3 to -20.0)	-21.1 (-27.1 to -15.1)	-22.8 (-28.9 to -16.7)	-18.7 (-24.4 to -13.0)

[1]Adjusted for child age and sex

[2]Adjusted for child age and sex, plus KS4 time-point variables (persistent school absence, in receipt of free school meals, school mobility, IDACI of residential neighbourhood)

[3]Adjusted for child age and sex, plus early-life[maternal and SEP] variables (maternal age at delivery, education, partner status, housing tenure, financial difficulties, smoking, depression, social support)

[4]Adjusted for child age and sex, plus KS4 and early life variables

For peer review only

Table G: Educational attainment, absence, and special educational needs by care status excluding those with a disability (n=9506)

Outcome	Care status during KS4	Model 1[1] (Age and Sex)	Model 2[2] (KS4 variables)	Model 3[3] (Early-life variables)	Model 4[4] (Fully adjusted)
		OR (95% CI)	OR (95% CI)	OR (95% CI)	OR (95% CI)
5 A*-C GCSEs inc. Eng. & Maths	Not CIN or CLA	Ref	Ref	Ref	Ref
	CIN (not CLA)	0.07 (0.02-0.25)	0.12 (0.03-0.43)	0.13 (0.04-0.45)	0.18 (0.05-0.65)
	CLA	0.17 (0.07-0.43)	0.19 (0.07-0.49)	0.28 (0.11-0.73)	0.29 (0.11-0.76)
		Coeff (95% CI)	Coeff (95% CI)	Coeff (95% CI)	Coeff (95% CI)
Capped percentage score	Not CIN or CLA	Ref	Ref	Ref	Ref
	CIN (not CLA)	-25.7 (-31.7 to -19.6)	-12.9 (-18.5 to -7.4)	-19.0 (-24.5 to -13.4)	-10.7 (-16.0 to -5.5)
	CLA	-28.6 (-34.2 to -23.0)	-24.6 (-29.7 to -19.5)	-22.8 (-28.0 to -17.6)	-20.8 (-25.7 to -16.0)

[1]Adjusted for child age and sex

[2]Adjusted for child age and sex, plus KS4 time-point variables (persistent school absence, in receipt of free school meals, school mobility, IDACI of residential neighbourhood)

[3]Adjusted for child age and sex, plus early-life[maternal and SEP] variables (maternal age at delivery, education, partner status, housing tenure, financial difficulties, smoking, depression, social support)

[4]Adjusted for child age and sex, plus KS4 and early life variables

For peer review only

Supplementary Figure 1: Partner, child and teacher questionnaire response rates by child social care status

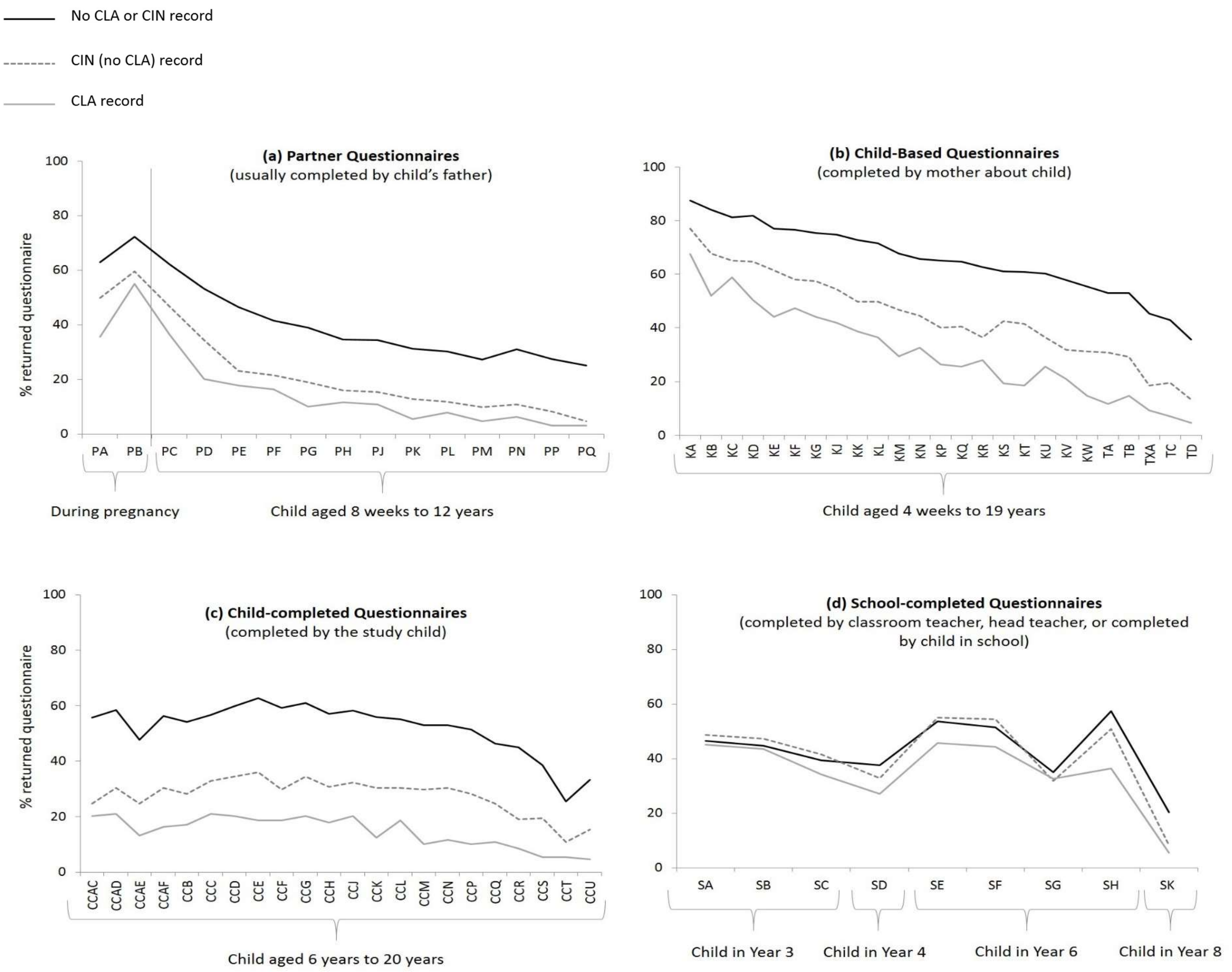

Early-life adversity, contact with children's social care services, and educational outcomes at age 16 years

Teyhan, Boyd, Wijedasa and Macleod

Supplementary Text - Additional details on some variables

- **Special Educational Needs (SEN)**

School Action (SA) - used when there is evidence that a child is not making progress at school and there is a need for action to be taken to meet learning difficulties. This can include involvement of extra teachers, use of different learning materials, special equipment or a different teaching strategy.

School Action Plus (SA+) - used where SA has not been able to help the child make adequate progress. The school has sought external services from the local education authority (LEA), the local health authority, or social services to help the child make adequate progress (e.g. speech and language therapist, educational psychologist etc.).

Statement of SEN - if the additional help provided by SA+ is not enough then the child's school or parents can apply to the LEA for a Statutory Assessment of the child's SEN in order to try and obtain a statement of SEN. The 'statement' is a document which sets out a child's SEN and any additional help that the child should receive. A Statement is normally made when all the educational provision required to meet a child's needs cannot reasonably be met by the resources within a child's school at SA+.

- **Early-life, maternal and family characteristics**

The following maternal, family and socioeconomic measures were reported by the mothers during their pregnancy with the study child via postal questionnaires. The categories for each variable are given in parenthesis following the variable name.

Highest educational qualification (university degree/A level; O level; vocational/none)

For peer review only

Early-life adversity, contact with children's social care services, and educational outcomes at age 16 years

Teyhan, Boyd, Wijedasa and Macleod

Financial Difficulties (quartiles of score with range 0–40, where 0 is no financial difficulties)

Housing tenure (owned/mortgaged; private rent; council rent; other)

Partner status (husband; live with partner; do not live with partner/no partner)

Smoking during pregnancy (no; yes)

Low social support - measured by response to 10 items with a low score defined as being in the bottom 10% (no; yes).

Maternal depression - measured by the Edinburgh Postnatal Depression Scale. Although this measure was originally designed for use with postnatal women, none of the 10 items is specific to this period and it has been validated for use at other times; it was chosen as it does not contain somatic items that could confound normal symptoms in pregnancy with depression (27). Quartiles were derived.

- **Proxy measures of socio-economic position when child aged 16**

The following measures were obtained from the National Pupil Database.

Child in receipt of free school meals (FSM) - eligibility is based on low parental income (28) (no; yes)

Child's residential neighbourhood deprivation - measured by the Income Deprivation Affecting Children Index (IDACI) (29), which gives the proportion of children (<0-15yrs) in a neighbourhood (lower super output area, average population 1500) who live in a low income family (<10%; 10 to <20%; 20 to <30%; 40%+).

For peer review only

STROBE Statement—checklist of items that should be included in reports of observational studies

	Item No	Recommendation	Page No
**Title and abstract**	1	(*a*) Indicate the study's design with a commonly used term in the title or the abstract	1,2
		(*b*) Provide in the abstract an informative and balanced summary of what was done and what was found	2
**Introduction**			
Background/rationale	2	Explain the scientific background and rationale for the investigation being reported	4-5
Objectives	3	State specific objectives, including any prespecified hypotheses	5
**Methods**			
Study design	4	Present key elements of study design early in the paper	5-6
Setting	5	Describe the setting, locations, and relevant dates, including periods of recruitment, exposure, follow-up, and data collection	5-6
Participants	6	(*a*) *Cohort study*—Give the eligibility criteria, and the sources and methods of selection of participants. Describe methods of follow-up<br>*Case-control study*—Give the eligibility criteria, and the sources and methods of case ascertainment and control selection. Give the rationale for the choice of cases and controls<br>*Cross-sectional study*—Give the eligibility criteria, and the sources and methods of selection of participants	5
		(*b*) *Cohort study*—For matched studies, give matching criteria and number of exposed and unexposed<br>*Case-control study*—For matched studies, give matching criteria and the number of controls per case	N/A
Variables	7	Clearly define all outcomes, exposures, predictors, potential confounders, and effect modifiers. Give diagnostic criteria, if applicable	6-8
Data sources/ measurement	8*	For each variable of interest, give sources of data and details of methods of assessment (measurement). Describe comparability of assessment methods if there is more than one group	5-8
Bias	9	Describe any efforts to address potential sources of bias	6,8,9
Study size	10	Explain how the study size was arrived at	5
Quantitative variables	11	Explain how quantitative variables were handled in the analyses. If applicable, describe which groupings were chosen and why	6-8
Statistical methods	12	(*a*) Describe all statistical methods, including those used to control for confounding	8-9
		(*b*) Describe any methods used to examine subgroups and interactions	N/A
		(*c*) Explain how missing data were addressed	8
		(*d*) *Cohort study*—If applicable, explain how loss to follow-up was addressed<br>*Case-control study*—If applicable, explain how matching of cases and controls was addressed<br>*Cross-sectional study*—If applicable, describe analytical methods taking account of sampling strategy	N/A
		(*e*) Describe any sensitivity analyses	9

Continued on next page

For peer review only - http://bmjopen.bmj.com/site/about/guidelines.xhtml

**Results**			
Participants	13*	(a) Report numbers of individuals at each stage of study—eg numbers potentially eligible, examined for eligibility, confirmed eligible, included in the study, completing follow-up, and analysed	Fig 1
		(b) Give reasons for non-participation at each stage	Fig 1
		(c) Consider use of a flow diagram	Fig 1
Descriptive data	14*	(a) Give characteristics of study participants (eg demographic, clinical, social) and information on exposures and potential confounders	Table 1
		(b) Indicate number of participants with missing data for each variable of interest	Table A
		(c) *Cohort study*—Summarise follow-up time (eg, average and total amount)	N/A
Outcome data	15*	*Cohort study*—Report numbers of outcome events or summary measures over time	Table 2
		Case-control study—Report numbers in each exposure category, or summary measures of exposure	N/A
		Cross-sectional study—Report numbers of outcome events or summary measures	N/A
Main results	16	(*a*) Give unadjusted estimates and, if applicable, confounder-adjusted estimates and their precision (eg, 95% confidence interval). Make clear which confounders were adjusted for and why they were included	Table 2
		(*b*) Report category boundaries when continuous variables were categorized	N/A
		(*c*) If relevant, consider translating estimates of relative risk into absolute risk for a meaningful time period	N/A
Other analyses	17	Report other analyses done—eg analyses of subgroups and interactions, and sensitivity analyses	10
**Discussion**			
Key results	18	Summarise key results with reference to study objectives	15
Limitations	19	Discuss limitations of the study, taking into account sources of potential bias or imprecision. Discuss both direction and magnitude of any potential bias	15,17
Interpretation	20	Give a cautious overall interpretation of results considering objectives, limitations, multiplicity of analyses, results from similar studies, and other relevant evidence	17,18
Generalisability	21	Discuss the generalisability (external validity) of the study results	16
**Other information**			
Funding	22	Give the source of funding and the role of the funders for the present study and, if applicable, for the original study on which the present article is based	19

*Give information separately for cases and controls in case-control studies and, if applicable, for exposed and unexposed groups in cohort and cross-sectional studies.

Note: An Explanation and Elaboration article discusses each checklist item and gives methodological background and published examples of transparent reporting. The STROBE checklist is best used in conjunction with this article (freely available on the Web sites of PLoS Medicine at http://www.plosmedicine.org/, Annals of Internal Medicine at http://www.annals.org/, and Epidemiology at http://www.epidem.com/). Information on the STROBE Initiative is available at www.strobe-statement.org.

For peer review only
